# In Vivo Administration of a JAK3 Inhibitor during Acute SIV Infection Leads to Significant Increases in Viral Load during Chronic Infection

**DOI:** 10.1371/journal.ppat.1003929

**Published:** 2014-03-06

**Authors:** Yoshiaki Takahashi, Siddappa N. Byrareddy, Christina Albrecht, Markus Brameier, Lutz Walter, Ann E. Mayne, Paul Dunbar, Robert Russo, Dawn M. Little, Tara Villinger, Ladawan Khowawisetsut, Kovit Pattanapanyasat, Francois Villinger, Aftab A. Ansari

**Affiliations:** 1 Department of Pathology and Laboratory Medicine, Emory University School of Medicine, Atlanta, Georgia, United States of America; 2 Primate Genetics Laboratory, German Primate Center, Leibniz-Institute for Primate Research, Göttingen, Germany; 3 Department of Immunology, Faculty of Medicine Siriraj Hospital, Mahidol University, Bangkok, Thailand; 4 Office for Research and Development, Faculty of Medicine Siriraj Hospital, Mahidol University, Bangkok, Thailand; 5 Division of Pathology, Yerkes National Primate Research Center, Emory University, Atlanta, Georgia, United States of America; Vaccine Research Center, United States of America

## Abstract

The studies reported herein are the first to document the effect of the in vivo administration of a JAK3 inhibitor for defining the potential role of NK cells during acute SIV infection of a group of 15 rhesus macaques (RM). An additional group of 16 MHC/KIR typed RM was included as controls. The previously optimized in vivo dose regimen (20 mg/kg daily for 35 days) led to a marked depletion of each of the major NK cell subsets both in the blood and gastro-intestinal tissues (GIT) during acute infection. While such depletion had no detectable effects on plasma viral loads during acute infection, there was a significant sustained increase in plasma viral loads during chronic infection. While the potential mechanisms that lead to such increased plasma viral loads during chronic infection remain unclear, several correlates were documented. Thus, during acute infection, the administration of the JAK3 inhibitor besides depleting all NK cell subsets also decreased some CD8^+^ T cells and inhibited the mobilization of the plasmacytoid dendritic cells in the blood and their localization to the GIT. Of interest is the finding that the administration of the JAK3 inhibitor during acute infection also resulted in the sustained maintenance during chronic infection of a high number of naïve and central memory CD4^+^ T cells, increases in B cells in the blood, but decreases in the frequencies and function of NKG2a^+^ NK cells within the GIT and blood, respectively. These data identify a unique role for JAK3 inhibitor sensitive cells, that includes NK cells during acute infection that in concert lead to high viral loads in SIV infected RM during chronic infection without affecting detectable changes in antiviral humoral/cellular responses. Identifying the precise mechanisms by which JAK3 sensitive cells exert their influence is critical with important implications for vaccine design against lentiviruses.

## Author Summary

In efforts to define the potential role of innate immune effector mechanisms in influencing the course of SIV infection during the acute infection period, our lab utilized the in vivo daily administration of 20 mg/kg orally of a compound called Tofacitinib (a Janus kinase 3 inhibitor) to a group of 15 rhesus macaques starting at day −6 and until day 28 post intravenous SIVmac239 infection. An additional group of 16 similarly SIV infected rhesus macaques served as a placebo control. This drug targets the JAK/STAT pathway that is utilized by cells including the NK cell lineage, a major cell of the innate immune system. The dosage utilized was based on extensive previous PK studies that resulted in a marked depletion of the NK cells. Of interest while such drug administration had no effect on plasma viral loads during acute infection, such drug administration led to significant increases in plasma and gastro-intestinal tissues (GIT) viral loads during chronic infection. A series of phenotypic/functional studies were performed to determine the mechanisms for this delayed effect and the correlates identified. These data are the first to document the effect of JAK-3 inhibitor during acute SIV infection with implications for HIV vaccine design.

## Introduction

The general consensus opinion is that events during the acute infection period following pathogenic lentiviral infection of humans and nonhuman primates dictate not only the levels of peak viremia but also the rate of disease progression [1]–[3]. This view is based on the observation that diverse viral loads and rates of disease progression are noted following acute infection of rhesus macaques with an aliquot of the same pool of SIV and by the same route of infection. Since adaptive immune responses take time to develop, it thus seems logical that innate immune effector mechanisms must play a major role in influencing the outcome during acute viral infections [4]–[6]. Among the innate immune hematopoietic effector cells that could contribute in this regard are the natural killer (NK) cells. The phenotypic characteristics, differentiation, development and function of the NK cell lineage have been a subject of study for the past several decades [7]–[19]. It is reasonably clear that there is both phenotypic and functional heterogeneity of this cell lineage some of which is associated with the tissue and organ in which this cell lineage resides [16], [17], [20]–[22]. It is also fair to state that our previously held view that this cell lineage only performs killing function and has no immunological memory has been over simplistic. This cell lineage is now known to require self-MHC education, become licensed, possess immunological memory, manifest regulatory function (NKregs) and even contribute to tissue regeneration [21], [23]–[43].

The precise in vivo role of the NK cell lineage in influencing acute as well as chronic lentiviral infection continues to be defined. One practical and definitive method to study the in vivo role of this cell lineage involves the depletion of this cell lineage using a variety of biological and chemical agents and, although effective, they each have limitations that we have summarized elsewhere [44]. Use of a small molecule inhibitor such as Tofacitinib, a Janus Kinase 3 (JAK3) inhibitor that can deplete NK cells in vivo was reasoned by our lab to be a very useful tool because we could reliably monitor the plasma levels of this drug in vivo [45]. It is important to note that while earlier studies appeared to suggest a high degree of JAK3 inhibition specificity by Tofacitinib, later studies appeared to suggest that the molecule also inhibited JAK2 and JAK1 but inhibition of these latter 2 kinases were dose dependent [46]–[50]. The data presented herein thus need to be evaluated with such findings in mind. Success of the initial studies [45] in depleting NK cells in vivo prompted us to conduct a more detailed in vitro and in vivo pharmacokinetic study of the JAK3 inhibitor (Tofacitinib) in rhesus macaques to define an optimal dosing strategy that would preferentially deplete the NK cell lineage [44]. This identified dosing schedule was utilized and shown indeed to preferentially deplete NK cells in vivo in 6 rhesus macaques chronically infected with SIV [44]. A transient increase in plasma viral RNA and cellular pro-viral DNA loads were noted. These initial findings led us to conduct a more detailed study of the effect of this JAK3 inhibitor administration during acute SIV infection in a cohort of 15 rhesus macaques (initially 16 but one was removed, see below) and for purposes of comparison a control cohort of age matched 16 additional similarly SIV infected rhesus macaques. Results of these studies has led to the observation that effective depletion of a high frequency of NK cells was achieved by the administration of this JAK3 inhibitor in all tissues examined during acute infection. Such depletion while not appreciably influencing plasma viral loads during acute infection, led to a significant increase in pro-viral DNA loads in the gastro-intestinal tract (GIT) during this same time period. Surprisingly however, the administration of the JAK3 inhibitor during acute infection promoted a significant increase in both the plasma and GIT viral load during chronic infection. These findings have important clinical implications since this JAK3 inhibitor is FDA approved for use in patients with a number of autoimmune diseases including rheumatoid arthritis [51]–[54]. In the studies reported herein, the potential mechanisms by which the in vivo administration of a JAK3 inhibitor that includes NK cell depletion during the acute phase mediates its effect on both the plasma and GIT during chronic infection despite relatively rapid replenishment of a significant number of the NK cell lineage in the blood are discussed.

## Materials and Methods

### Animals and Source of Blood & Colo-rectal Biopsy Samples

Juvenile to adult male rhesus macaques (*Macaca mulatta*) of Indian origin were used for the studies reported herein. The animals were all housed at the Yerkes National Primate Research Center (YNPRC) of Emory University (Atlanta, GA) and were maintained according to the guidelines of the Committee on the Care and Use of Laboratory Animals of the Institute of Laboratory Animal Resources, National Research Council and the Department of Health and Human Service guideline titled Guide for the Care and Use of Laboratory Animals. All studies were reviewed and approved by the Emory University IACUC. Three groups of rhesus macaques comprised the present study. The basic protocol is illustrated in Figure S1. Group 2 (n = 15) was administered the JAK3 inhibitor orally at 20 mg/kg daily for 35 days starting day −6 prior to infection. Group 1 (n = 16) was age matched and infected with SIV similarly to Group 2 and served as untreated controls monitored in parallel. Group 3 (n = 4) was administered the JAK3 inhibitor and infected according to the same protocol as Group 2 except each monkey in addition was also administered a loading dose of a “primatized” anti-IL15 mAb (20 mg/kg) on day −3 followed by bi-weekly dose of 10 mg/kg for a total of 8 weeks (optimized protocol courtesy of Dr. Afam Okoye, Oregon Health Sciences University, Beaverton, OR). One of the original 16 monkeys that received the JAK3 inhibitor (Group 2) was eliminated from the study due to causes unrelated to the JAK3 inhibitor and/or SIV infection. Thus, group 2 consisted of 15 animals. Each monkey was infected intravenously with 200 TCID50 of SIVmac239 on day 0. Viral loads were monitored in plasma samples every week for 4 weeks, every other week for 8 weeks and monthly thereafter, using bDNA quantitation on aliquots of EDTA-plasma by Siemens Inc. (Berkeley, CA). Cellular pro-viral DNA loads and polychromatic phenotypic analyses of subsets of cells in aliquots of mononuclear cells from colo-rectal biopsy tissue samples were performed on cells isolated as described elsewhere [44]. Viral loads were expressed as number of copies/ng DNA with a sensitivity of detection of 1 copy/ng DNA [55]. Blood and colo-rectal biopsy samples for phenotypic and functional analyses were obtained prior to (3 baseline samples) and at weekly intervals until 4 weeks, bi-weekly until 8 weeks and monthly intervals thereafter. Finally, data on the effect of the JAK-3 inhibitor (administered the same dose) alone on the absolute numbers of CD4, CD8, NKG2a^+^ and pDC's in PBMC samples and GIT biopsy samples from 6 rhesus macaques from a previous study were included to distinguish differences if any of the effect of the JAK3 inhibitor alone as compared with SIV alone or JAK3 inhibitor treated SIV infected animals.

### Ethics Statement

All animals were born and maintained at the YNPRC of Emory University in accordance with the rules and regulations of the Committee on the Care and Use of Laboratory Animal Resources. The animals were fed monkey diet (Purina) supplemented daily with fresh fruit or vegetables and water ad libitum. Additional social enrichment including the delivery of appropriate safe toys were provided and overseen by the Yerkes enrichment staff and animal health was monitored daily and recorded by the animal care staff and veterinary personnel, available 24/7. Monkeys were caged in socially compatible same sex pairs to facilitate social enhancement and wellbeing. Monkeys showing signs of sustained weight loss, disease or distress were subject to clinical diagnosis based on symptoms and then provided either standard dietary supplementation analgesics and/or chemotherapy. Monkeys whose symptoms could not be alleviated using standard dietary supplementation, analgesics and/or chemotherapy were humanely euthanized using an overdose of barbiturates according to the guidelines of the American Veterinary Medical Association. The studies reported herein were performed under IACUC protocol #2001186 “Innate immunity in SIV infection” which was reviewed and approved by the Emory University IACUC. It has been assigned the IACUC protocol number “YER-2001186-082414GA”. The YNPRC has been fully accredited by the Association for Assessment and Accreditation of Laboratory Animal Care International since 1985. All experiments were reviewed and approved by the Emory biosafety review Committee.

### JAK3 Inhibitor

The JAK3 inhibitor (Tofacitinib) was purchased from LC Laboratories (Woburn, MA). For in vivo administration, the compound Tofacitinib (313 m.w.) was prepared by first dissolving it in DMSO and then in methanol to obtain a 2 mM solution in 0.5% methylcellulose. An appropriate amount was then incorporated in peanut butter based on the weight of the individual monkey and administered at a dose of 20 mg/kg orally for a period of 35 days. Quantitation of plasma levels of the Tofacitinib was performed using a reverse phase-HPLC with MS/MS with a detection level sensitivity of 2.5 ng/ml [56]. We have reported the in vivo safety of this drug in rhesus macaques elsewhere [44]. The biological effect of JAK3 on the immune system in vivo was monitored by Western Blot analyses of ratios of phosphorylated and non-phosphorylated STAT-5 (total STAT-5 and *p-STAT-5) in lysates of an aliquot of PBMCs as described previously [44]. This dosing schedule does not have any detectable effect on TCR signaling.

### The Administration of the Anti-IL15 monoclonal Antibody (mAb)

As noted below, the in vivo administration of the JAK-3 inhibitor at the doses described did not completely deplete the NK cells during acute infection. We reasoned that this failure to completely deplete the NK cells might have resulted in our failure to observe any significant effect on plasma viral loads during the acute infection period. This prompted us to study the effect of supplementing the JAK3 inhibitor administration with a unique “primatized” anti-IL15 mAb (NIH non-human primate reagent resources, Beth Israel Hospital, Boston, MA). A group of 4 rhesus macaques (Group 3) were thus administered the same dose and schedule of the JAK3 inhibitor as described above but, in addition, received a loading dose of 20 mg/kg of the anti-IL15 mAb intravenously on day −2 and then 10 mg/Kg of this same mAb intravenously twice a week for a total of 8 weeks (protocol established by the Picker lab, University of Oregon, Portland, OR).

### Polychromatic Flow Cytometric Analyses

The frequencies of subsets of mononuclear cells in aliquot of blood samples and on cells isolated from pools of GI tissue biopsies was performed using panels of monoclonal antibody reagents with a focus on the analyses of NK cells and its subsets [16]. In brief, the PBMC's were isolated from heparinized blood and the mononuclear cells isolated from pools of colo-rectal biopsies by techniques outlined elsewhere [44]. Absolute values of each cell lineage in the blood were calculated from CBC's that were performed on an aliquot of each blood sample. The cell lineages identified from the GIT was expressed as the number of cell per µg of tissue utilized. A battery of rhesus macaque reactive commercially purchased monoclonal antibody reagents conjugated with a variety of fluorochromes were used for polychromatic flow cytometric analysis of lymphoid cells utilizing a LSR-II flow cytometer (BD Immunocytometry Division, Mountain View, CA). Appropriate panels of the monoclonal antibodies were used to identify subsets of T cells, B cells, NK cells, monocytes, myeloid (mDC) and plasmacytoid dendritic cells (pDC) as described elsewhere [44], [63]. The mAbs were purchased from BD Biosciences (San Jose, CA) and included Alexa700- and PE-Cy5-anti-CD3 (clone SP34-2), FITC-, PE-and Pac Blue-anti-CD8 (clones SK1 and clone RPA-T8), PerCP- and Pac Blue-anti-CD4 (clone L200), FITC-anti-CD14 (clone M5E2), FITC- and APC-Cy7-anti-CD16 (clone 3G8), APC-anti-CD20 (clone 2H7), PE-Cy7-anti-CD56 (clone NCAM16.2), PE-Cy5-anti-CD95 (clone DX2), FITC-anti-CD197 (clone 3D12), FITC- and Per-CP5.5-anti-HLA-Dr (clone G46-6), PE-Cy5-anti-CD45 (clone TU116), PE-Cy5-anti-CD107a (clone LAMP-1), FITC-Lin- (cocktail), APC-CD11c (clone S-HCL-3), APC-anti-CD123 (clone 7G3), PE-CD21 (clone B-ly4), and FITC-CD27 (clone M-T271). The mAb purchased from Beckman Coulter (Brea, CA) included PE-anti-NKG2a (clone Z199), PR-TR-anti-CD28 (clone CD28-2) and PE-NKp44 (clone Z231). The mAb Alexa647-anti-IL-17A (clone eBio64DEC17) was purchased from eBioscience (San Diego, CA) and the mAb APC-anti-IFN-α (clone LT27:295) and PE/APC-anti-CD25 (clone 4E3) was purchased from Miltenyi (Auburn, CA). Select studies utilized PE-anti-Ki-67 and PE-conjugated p11c-Mamu-A01*-MHC class I tetramer (courtesy The NIH tetramer core facility, Emory University School of Medicine, Atlanta, GA). A minimum of 40,000 events was analyzed for each cell lineage except for mDC/pDC which required a minimum of 100,000 events. The data obtained was analyzed using the FlowJo software (Treestar, Ashland, OR) software. Phenotypically NK cells were defined as cells that were CD3^−^, CD8^+^, NKG2a^+^ which includes the 4 major subsets of NK cells as described elsewhere [16]. Representative profiles of the gating strategies utilized to quantitate the frequencies and absolute numbers of CD4^+^ and CD8^+^ T cells and their naïve, central memory and effector memory subsets; NK cells and its subsets: and plasmacytoid and myeloid dendritic cells are illustrated in Figure S2, S3 & S4, respectively.

### Genetic Typing of the Rhesus Macaques

Total RNA was isolated from the PBMC of each of the 31 monkeys, reverse transcribed and the cDNA was subjected to Roche/454 deep sequencing of MHC-class I and KIR gene transcripts, essentially as previously described [57], [58]. The MHC/KIR typing results for each of the 31 monkeys are provided in Table S1 A & B. As noted, the monkeys belonging to Groups 1 and 2 included a total of 31 Mamu-A*001^+^ monkeys primarily because this would allow us to determine the effect of JAK3 administration on the virus specific cellular responses using the p11C SIVgag peptide loaded tetramer. In addition, 30/31 of the monkeys chosen were B*008- and all 31 were B*017^−^ to minimize and exclude monkeys that have a high probability to spontaneously control SIV viremia. The 4 monkeys receiving the combined anti-IL15 and JAK3 inhibitor were Mamu-A*001^+^, B*008^−^, B*017^−^.

### Anti-SIV Antibody ELISA Titers, Neutralizing Antibodies and ADCC Assays

Aliquots of plasma samples from each of the 35 monkeys in this study were assayed for SIV specific antibodies using our lab standardized ELISA [55]. SIV neutralizing antibody analyses were performed using the TZM-bl assay [59]. Heat inactivated (56°C, 30 min) plasma were also analyzed for ADCC activity essentially as described [60] using the rCD16 KHYG-1 and the sLTR-Luc-CEM.NKR-CCR5 cell lines as effector and target cells, respectively. These cell lines were a kind gift from Dr. David T. Evans (current address: The Wisconsin National Primate Research Center, Madison, WI).

### Functional NK Assay

An aliquot of the PBMC sample was analysed for NK cell functional activity by co-culturing the PBMC in vitro with HLA class I-devoid K562 or 721.221 cells at a ratio of 10∶1 for 6 hrs at 37°C in a humidified 7% CO_2_ incubator in the presence of Brefeldin-A (5 µg/ml, Sigma-Aldrich), Monensin (6 µg/ml, Golgi-Stop, BD Biosciences) and anti-CD107a (clone LAMP1, BD Biosciences). Media throughout consisted of RPMI 1640 supplemented with 10% fetal calf serum, 2 mM L-glutamine, 100 units/ml penicillin and 100 µg/ml streptomycin. Each assay included an aliquot of the same PBMC cultured alone (negative control) and an aliquot of the same PBMC incubated with 1.25 µg/ml phorbol-12-myristate 13-acetate (PMA, Sigma-Aldrich) and 0.25 µg/ml ionomycin (Sigma Aldrich) as a positive control. Only experiments in which the negative control gave values of <10% value and there was a >3-fold increase between the negative and positive control were considered valid. After the co-culture, the cells were stained for viability using the Aqua LIVE/DEAD (Invitrogen) and stained with anti-CD3, CD8α, NKG2a, CD16 and CD56 antibodies. The cells were washed and re-suspended in the FACS buffer and analysed using the LSRII flow cytometer. A minimum of 100,000 events were analysed and the frequency of the gated population of CD3^−^, CD8^+^, NKG2a^+^ CD16^+^ cells that were CD107a^+^ determined using FlowJo software (TreeStar, Ashland, OR). Data obtained in the experimental sample – the negative control was calculated and utilized to illustrate NK cell functional activity.

### NK Cell Susceptibility to Undergo Apoptosis

Preliminary studies by our lab had shown that there is a surge of NK cells in the peripheral blood during acute infection. This surge, however, is accompanied by a variable increased susceptibility of the NK cells to undergo CD95 mediated lysis [61]. We thus analyzed a subset of monkeys from the 2 groups for such NK cells susceptibility assay during the acute infection period. Briefly, immunobead purified 1×10^5^ NKG2a^+^ cells from the PBMC of the monkeys were incubated overnight in media containing 50 U/ml of recombinant human IL-2 alone (control) or media containing 1 µg/ml of soluble Fas-L (R&D Systems, Minneapolis, MN)+50 U/ml of recombinant human IL-2 (kind gift from Hoffman LaRoche, Nutley, NJ). The cultures were washed and stained for the NK cell surface markers as outlined above in addition to Annexin V. The cells were analyzed using a LSR-II flow cytometer and the net frequencies of NK cells that were Annexin V positive (after deduction of the IL-2 control) in the cultures containing Fas-L as compared with IL-2 alone calculated.

### Statistical Analyses

All the statistical comparison between groups was performed using multiple t-tests and the Wilcoxon-rank sum test was used to compare levels of peak viremia. Differences over time were analyzed using the Tukey's multiple comparison test and the Mann-Whitney ‘U’ test. All the reported *p*-values are based on two-sided testing and a value of p = <0.05 was considered statistically significant. All the statistical analysis was performed using Prism GraphPad Software (version 5, CA).

## Results

### Effect of JAK3 Inhibitor Administration on Plasma Viral Loads and Pro-Viral DNA Loads in the GIT

**Figure 1:**
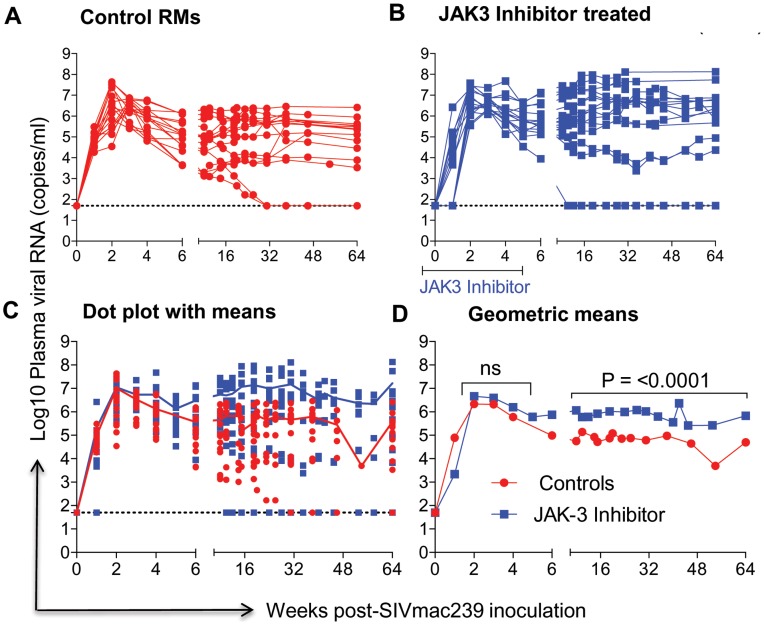
Aliquots of plasma samples from each of the 16 control monkeys and the 15 monkeys that received the JAK3 inhibitor were assayed for viral loads post SIVmac239 infection. The # of viral copies/ml of plasma from A) the control monkeys and B) the monkeys that received the JAK3 inhibitor are shown. The data in C) reflects the same data expressed as dot plots with means and D) illustrates these data as geometric means. (ns = non significant).

**Figure 2:**
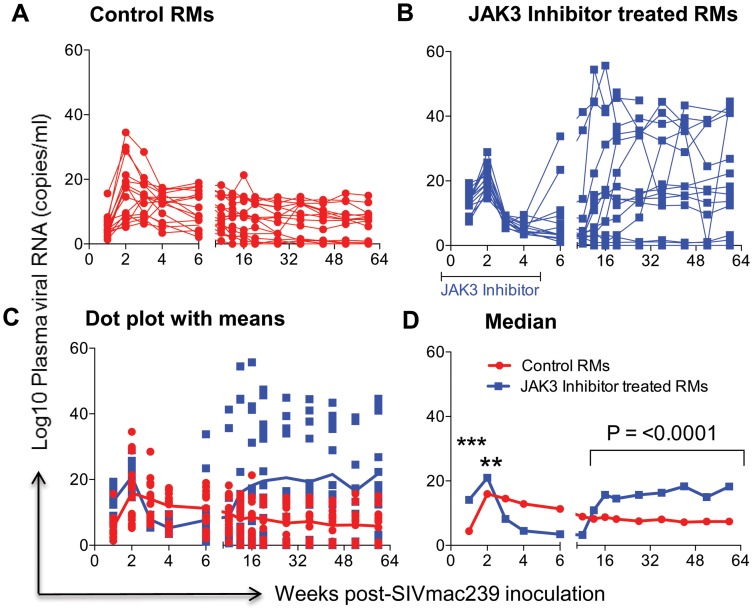
Aliquots of the mononuclear cells isolated from pools of GIT biopsies from the same animals as described under Figure 1 post SIVmac239 infection were used to isolate DNA and subjected to PCR using conserved SIVgag specific primer pairs. Data illustrated shows # copies/ng DNA from A) Control animals B) Animals that received the JAK3 inhibitor C) reflects the same data shown as dot plots with means and D) illustrates these data as Median values. The median values were chosen instead of Geometric means in this case because the formulae used to derive Geometric means cannot include negative or zero values. The symbols (***) represent p<0.0005 and (**) represents p<0.004 values.

The data on plasma RNA and gastro-intestinal tissue (GIT) pro-viral DNA loads as shown in Figures 1 & 2 are illustrated to highlight differences in viral loads noted during acute infection (0–6 weeks) and chronic infection (>12 weeks). As seen in Figure 1 A–D, there did not appear to be any statistical difference in the plasma viral loads between the control (Group 1) and the monkeys that received the JAK3 inhibitor (Group 2) during the acute infection period. However, following the acute infection period, there was a gradual increase in the plasma viral loads thereafter that reached statistical significance already during the chronic phase (p<0.0001) in the animals that received the JAK3 inhibitor during acute infection. Interestingly, 2 monkeys in the control group and 1 monkey in the JAK3 inhibitor group controlled plasma viremia spontaneously. Each of the 31 monkeys was also examined for gastro-intestinal tissue (GIT) associated pro-viral DNA levels as a function of time post infection. Mononuclear cells were isolated from pooled biopsies from each monkey at each time point and the DNA extracted from an aliquot and subjected to PCR analysis as described in the Methods section. As seen in Figure 2 A–D, there were several major differences in GIT pro-viral DNA loads in the 2 groups of animals. First of all, as early as one week post infection, there was a significant increase in GIT pro-viral DNA load in the JAK3 inhibitor group of monkeys as compared to controls (p<0.0005). There also appeared to be a significant difference in peak acute pro-viral DNA load in the JAK3 inhibitor group of monkeys (p<0.004). But, as noted above, there appeared to be marked sustained increased in the levels of pro-viral DNA load in the JAK3 inhibitor group during chronic infection (p<0.0001), a time period during which previous studies have documented nearly complete depletion of CD4^+^ T cells in the GIT. Thus, there appeared to be two waves of viremia within the GIT of the animals that received the JAK3 inhibitor. It is not clear at present whether these 2 waves of viremia were due to the replication of virus in 2 different cell lineages or the same. The precise cell lineage that contributes to such differences and sustained increases in pro-viral DNA load particularly during chronic infection remains unknown and was not studied because such data were unexpected and due to the paucity of cells that one can obtain from such biopsy samples.

### Effect of JAK3 Inhibitor Administration on the Frequencies of Subsets of Lymphoid Cells in the Blood

**Figure 3:**
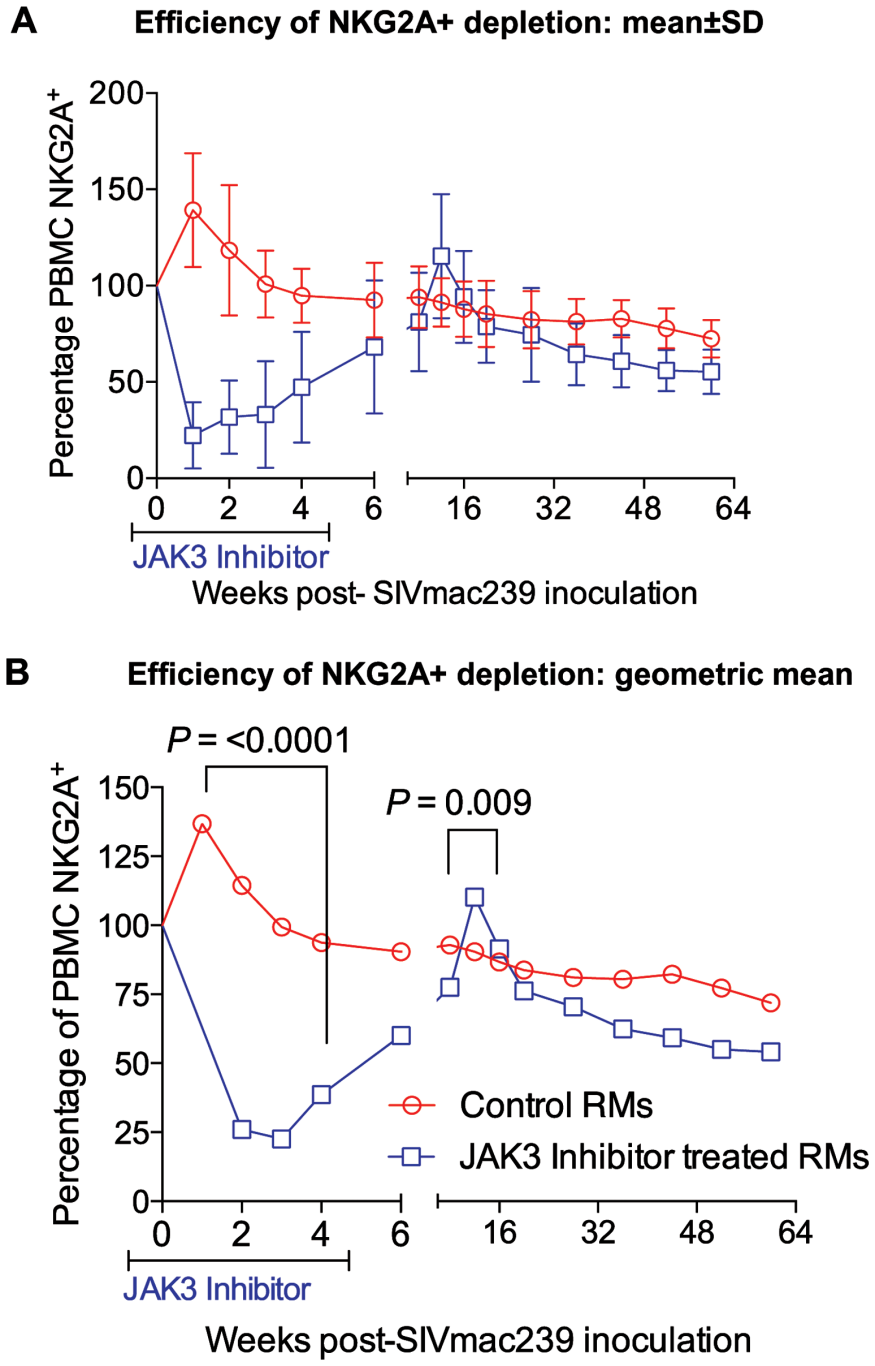
The efficiency (% change from a mean of 3 baseline values) of depletion in the absolute numbers of CD3^−^, CD8α^+^, NKG2a^+^ (total NK cells) in the PBMC of control and the animals that received the JAK3 inhibitor as a function of time post infection is illustrated. A) Shows the data with Means +/− SD and B) the Geometric means for the 2 groups of animals. The difference in the values at 2 weeks post infection in the 2 groups of animals was highly significant (p<0.0001).

**Figure 4:**
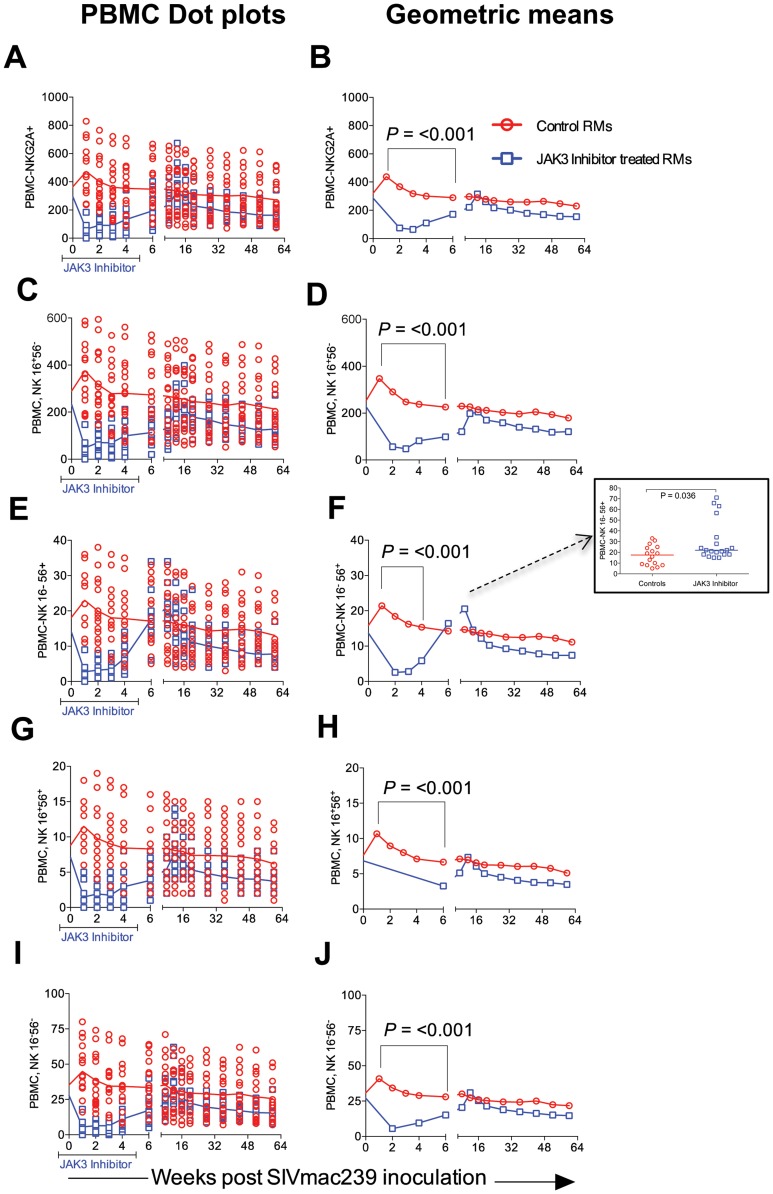
The absolute numbers of NK cells and its subsets within the PBMC of the 2 group of animals is illustrated as dot plots with means (A, C, E, G & I) and Geometric means (B, D, F, H & J) of total CD3^−^, CD8α^−^, NKG2a^+^ NK cells (A, B) and the CD16^+^, CD56^−^ cytolytic subset (C, D), the cytokine sythesizing subset CD16^−^, CD56^+^ (E, F), the CD16^+^, CD56^+^ (G, H) and the CD16^−^, CD56^−^ (I, J) subset. Please note the NK cell lineage that rebounds first (Inset Figure 4F).

The rationale for the in vivo administration of the JAK3 inhibitor was to deplete the NK cell lineage to assess the role of this cell lineage on influencing viral loads and rate of disease progression during acute SIV infection. PBMC samples were isolated and subjected to polychromatic flow cytometric analyses for a variety of cell lineages as described in the methods section. Figure 3 A, B summarizes the efficiency of the depletion of CD3−/CD8α^+^/NKG2a^+^ in the animals that received the JAK3 inhibitor. As seen, there appeared to be significant (p<0.0001) decrease with a mean of approximately 75% depletion of NK cells in the PBMC of these monkeys during the acute infection period. The kinetic changes in absolute numbers of total NK cells and NK cell subsets are illustrated in Figure 4 A–J. As seen, while there were increases in the frequencies of NK cells and their subsets during week 1 in the control animals, this was followed by a gradual decrease for each of the subsets during the chronic infection period (please note differences in scale for each subset). On the other hand (Figure 4 A–J), JAK3 inhibitor treatment was clearly very effective in depleting total NK cells (CD3^−^/CD8a^+^/NKG2a^+^) and each of the 4 major subsets of NK cells (CD16^+^/CD56^−^; CD16^−^/CD56^+^; CD16^+^/CD56^+^; and CD16^−^/CD56^−^) very rapidly with a nadir at 2 weeks that was sustained during the administration of the JAK3 inhibitor (p<0.001). Thereafter, there was a gradual increase to baseline levels followed by a decrease similar to that seen in the control animals that were lower but not statistically significant. The depletion of the NK cells prompted us to examine whether there was a difference in the sequence of emergence of the various subsets of NK cells that would provide clues as to the differentiation pattern of these subsets. The only major difference noted was the earlier emergence of the CD16^−^/CD56^+^ NK cell subset (p<0.036) suggesting that this cell subset precedes the differentiation of the predominant cytolytic CD16^+^/CD56^−^ subset (inset, Figure 4F).

**Figure 5:**
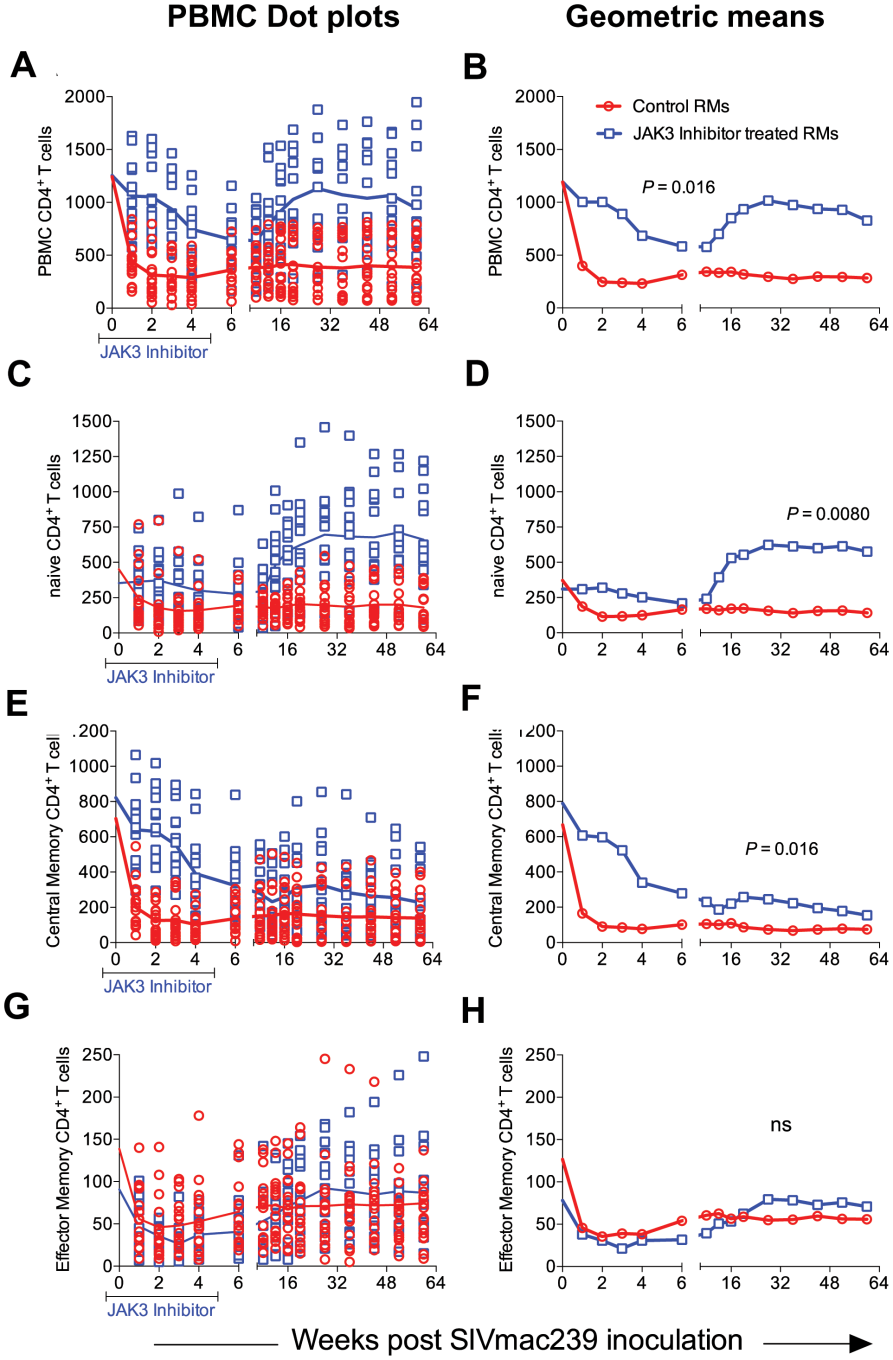
The absolute number of total (A & B), naïve (C & D) central memory (E & F) and effector memory (G & H) CD4^+^ T cells in the PBMCs of the 2 group of animals expressed as dot plots (A, C, E and G) and geometric means (B, D, F & H) is shown. While there was no significant difference in the numbers of effector memory CD4^+^ T cells between the 2 groups of animals, there was clearly a significant difference in the total (p<0.016), naïve (p<0.008) and central memory CD4^+^ T cells (p<0.016).

**Figure 6:**
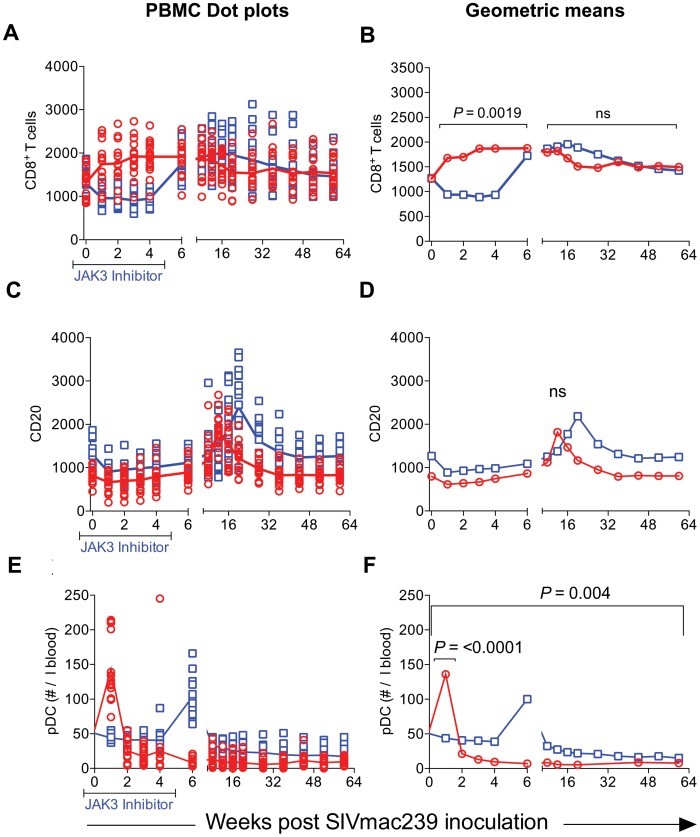
The absolute number of total CD8^+^ T cells (A & B), total B cells denoted as CD20 (C & D) and plasmacytoid dendritic cells denoted as pDC (E & F) in the PBMCs from the 2 groups of animals is illustrated as dot plots with means (A, C & E) and geometric means (B, D & F). The statistical differences are noted as either n.s. (Not significant) or with appropriate p values.

JAK3 inhibitor administration also had effects on several other hematopoietic cell lineages that are discussed individually. Thus, as seen in Figure 5 A–H, SIV infection led to the expected rapid depletion of total CD4^+^ T cells which includes depletion of the naïve, central and effector memory subsets in the control animals. As previously documented, the major target of this depletion appears to be the central memory CD4^+^ T cell subset (Figure 5 E, F) that was reduced from a mean of >600 to <200 cells/µl of blood within 2 weeks post infection. Of interest however, was the difference noted in the animals treated with the JAK3 inhibitor. Thus, while there was no difference in the kinetics of depletion of the effector memory CD4^+^ T cells among the 2 group of animals, there was clearly a “protective effect” on both the naïve (p<0.008) and central memory CD4^+^ T cell subsets (p<0.016). These differences accounted for the change seen in the kinetics of depletion of the total CD4^+^ T cells (Figure 5A/B, p<0.016). It is important to note the finding of the reconstitution of the naïve CD4^+^ T cell subset (Figure 5C & D) that was seen shortly after discontinuation of the JAK3 inhibitor administration which was also coincident with the increases seen in the plasma (Figure 1) and GIT viral loads (Figure 2) among the group 2 animals. The mechanisms for the protective effect of JAK3 inhibitor on the naïve and central memory CD4^+^ T cells are currently unknown and a subject of study. With regards to the CD8^+^ T cell lineage, as seen in Figure 6 A & B, while there was an increase in the absolute numbers of CD8^+^ T cells during acute infection in the control animals (p<0.0019), there was a significant decrease in the absolute numbers of CD8^+^ T cells in the JAK3 inhibitor group of animals (p<0.0019) during the same time period. Thereafter, there did not seem to be any detectable difference in the absolute number of CD8^+^ T or its subsets (naïve, central and effector memory, data not shown) in these 2 groups of animals suggesting that the decrease during the acute phase was likely due to the effect of the JAK3 inhibitor. Thus, the mechanisms responsible for differences in viral loads during chronic infection need to be interpreted within this context of mild and transient CD8 depletion. Next we examined the effect of JAK3 inhibitor on blood levels of B cells and pDCs. As seen in Figure 6 C & D, there appeared to be a slight decline in the absolute number of total B cells in both group of animals during the acute phase. However, during the chronic phase there appeared to be a significant increase in the absolute number of B cells in both groups of animals with a higher increase in the animals administered the JAK3 inhibitor. The peak increase in the 2 groups of animals was distinct. Thus, while the control group showed peak increases at 12–14 weeks p.i., the JAK3 inhibitor group showed peak increases at 16–20 week p.i., for reasons not clear at present. Subsets of B cells were also analyzed but each subset showed the same profile changes as noted for the total B cells (data not shown). We also examined the absolute number of plasmacytoid (pDCs) and myeloid dendritic cells (mDCs) in these 2 groups of animals. As noted previously, our lab was the first to show the rapid mobilization of pDCs in the blood during acute infection [64], which was also noted in the control animals (see Figure 6 E) and is thereafter rapidly reduced and stays reduced. On the other hand, this rapid increase of pDCs was not seen in the blood of the JAK3 inhibitor group of animals until right after the cessation of JAK3 inhibitor administration (Figure 6 E, F) with subsequent decreases in the blood. The increases during acute infection in the control animals and during chronic infection in the JAK3 inhibitor group were statistically significant (p<0.001 and p<0.004, respectively). These data suggest that the mobilization of the pDCs in the blood is sensitive to the JAK3 inhibitor. No significant changes, however, were noted in the mDCs during this same time period (data not shown).

### Effect of JAK3 Inhibitor Administration on the Frequencies of Subsets of Lymphoid Cells in the GIT

**Figure 7:**
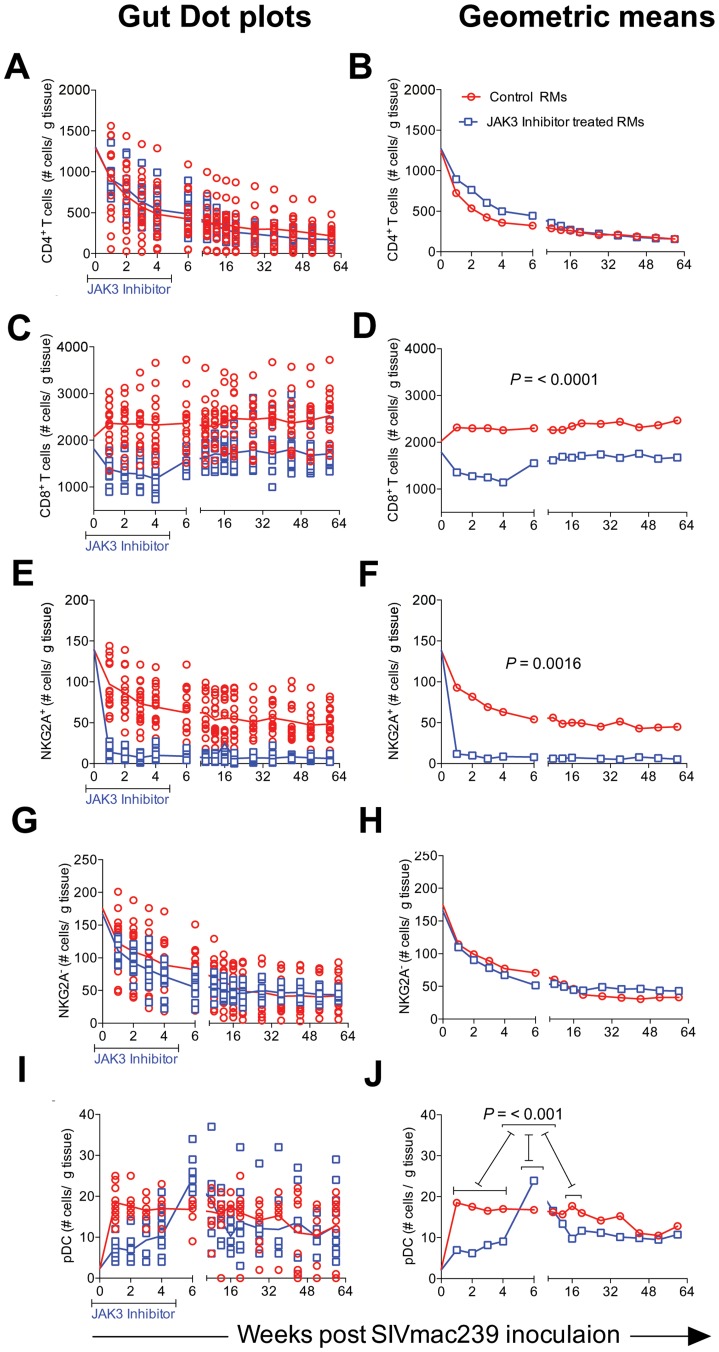
The frequencies of CD4^+^ T cells (A & B), CD8^+^ T cells (C & D), NKG2a^+^ cells (E & F), NKG2a^−^ cells (G & H) and plasmacytoid dendritic cells (I & J) within the mononuclear cells isolated from the GIT of the 2 groups of animals are illustrated as dot plots with means (A, C, E, G & I) and geometric means (B, D, F, H & J). Data are expressed as number of cells/gm of GIT biopsies as described in the methods section. Statistical evaluation of the data is included within each graph.

The limitations in the number of cells we could obtain from the colo-rectal biopsy tissues dictated that we conduct a more focused analysis of cell lineages with these cell samples as compared with blood samples. We thus elected to analyze the numbers of total CD4^+^ T cells and its subsets, CD8^+^ T cells, the frequencies of NKG2a^+^ cells (total NK cells), the frequencies of NKG2a^−^ cells that include the NKp44^+^ ILC cells [17] and pDCs. Instead of sampling each of the 16 animals in the control group and 15 animals of the JAK3 inhibitor group at each time point for each of the cell lineages, we chose to analyze samples from half of the animals from each group for one set of cell lineages and the other half for the rest of the cell lineages during one week and the reverse set of markers for the following week. Data as shown are expressed as number of each cell lineage within the pool of cells obtained based on the weight of the pooled GIT (# of cells/µg of tissue) utilized. As seen in Figure 7 A & B, there was a rapid depletion of CD4^+^ T cells in the GIT of both groups of animals with no significant differences noted and thus the peripheral blood profiles for CD4^+^ T cells did not match the profiles seen in the CD4^+^ T cells from the GIT. On the other hand, while there was an initial decrease in the frequencies of CD8^+^ T cells in the JAK3 inhibitor group of animals followed by replacement thereafter (Figure 7 C & D), the control animals did not show this decrease. In fact there was a gradual increase in the number of CD8^+^ T cells that was sustained during the chronic infection period and was significantly (p<0.0001) different from the JAK3 inhibitor group of animals. These data suggest that the JAK3 inhibitor markedly decreases both the blood and GIT CD8^+^ T cells. With regards to the NK cells, it was interesting to note that while there was a marked difference in the kinetics of decrease of the NKG2a^+^ cell lineage (p<0.0016) between the 2 groups of animals (Figure 7 E & F), there was no significant difference in the kinetic changes in the frequencies of the NKG2a- cell lineage (Figure 7 G & H). Thus, while there was complete depletion of the GIT NKG2a^+^ cells in the JAK3 inhibitor group that was sustained for a long period of time, the decrease of NKG2a^+^ cells was modest in the animals of the control group. Additionally, in a subset of samples analyzed, the frequencies of NKp44^+^ cells were almost completely depleted in both groups of animals. This cell lineage also never returned to detectable levels post infection (data not shown). These data suggest that the differences in the plasma and GIT viral loads between these 2 groups of animals appear to be correlated with the disappearance of the NKG2a^+^ and the NKp44^+^ cells within the GIT in the JAK3 inhibitor group. We also examined the frequencies of pDCs in the GIT because of their importance especially during acute infection as our lab had previously shown [62], [63]. Of interest was the observation that whereas there was a marked increase in the frequencies of pDCs rapidly following infection in the control group of animals (p<0.004), this increase was blunted during acute infection in the JAK3 inhibitor animals and the increase delayed until the cessation of JAK3 inhibitor administration (Figure 6 E & F) with a significant increase (p<0.001) thereafter, with a similar level of sustained increase in both groups of animals. These findings support the data previously documented by Reeves et al [64] that SIV infection leads to an accumulation of pDCs in the GIT.

### Functional Studies of NK Cells

**Figure 8:**
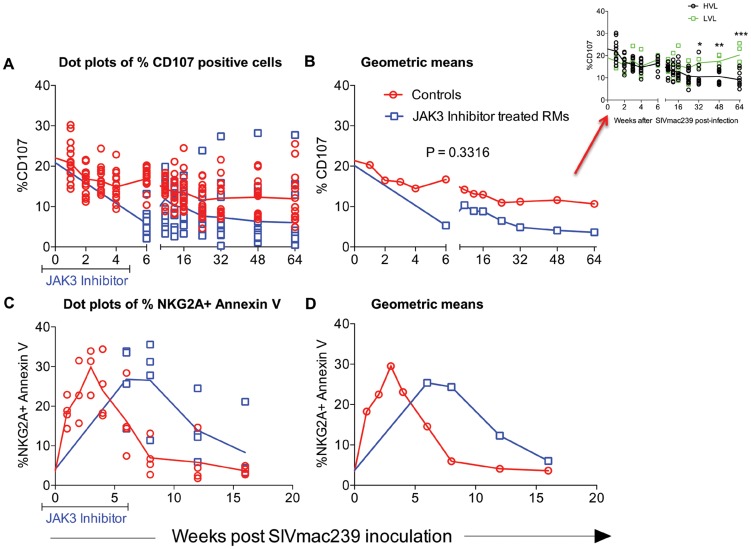
Functional assessment of NK cells in an aliquot of the PBMC from each of the 2 groups of animals was determined by measuring the frequencies of NK cells expressing CD107 upon co-culture with the 721.221 cell line as described in the methods section. The frequencies of CD107^+^ cells are expressed as dot plots with means (A) or geometric means (B). Aliquot of the same cells were also assessed for undergoing apoptosis in the presence and absence of soluble CD95 as described in the methods section. Data of the frequency of Annexin V expressing NKG2a^+^ cells is shown as dot plots (C) or geometric means (D). The differences in the re-emergence of NK cell function reflected as the frequencies of CD107+ NK cells in 4/16 control animals that spontaneously controlled plasma viremia (n = 2) and those with low viral loads (n = 2, LVL) as compared with those with high viral load (n = 12, HVL) is shown in the inset of Fig. 8 B.

Results from our previous studies had suggested that NK cells appear to be rapidly mobilized during acute infection but then they gradually decrease both numerically and functionally over time post SIV infection. Thus, while significant numbers of NK cells remain during chronic infection, the cytolytic subset (major subset) lose their function [4], [65]–[67]. In addition, these NK cells become highly susceptible to CD95 induced apoptosis [61]. In efforts to define the role of the JAK3 inhibitor on these 2 functions of NK cells, aliquots of PBMC from the 2 groups of animals were assessed for both NK cell cytolytic function and susceptibility to apoptosis as described in the methods section. It is to be noted that due to the effect of the JAK3 inhibitor on depletion of NK cells, samples from the monkeys that received the JAK3 inhibitor could only be assessed for function following cessation of JAK3 inhibitor administration. As seen in Figure 8 A & B, there is a gradual reduction of NK cell functional activity from baseline values as a function of time post infection in each of the 16 control monkeys and 15 JAK3 inhibitor group of animals. Interestingly, it was noted that 2/16 control animals that were spontaneous controllers of viremia and 2/16 that had relatively lower viral load together showed a marked difference in the kinetics of return of NK cell function as compared with the remaining 12/16 animals (p<0.0001) suggesting a correlation between control of viral loads and reconstitution of NK cell emergence and function (see Figure 8 B, inset). The NK cell depleted animals, on the other hand showed low NK cell function shortly after cessation of JAK3 inhibitor administration and while some variability in return of function was noted, the function never returned to baseline values. Similarly, when the NK cells were assessed for susceptibility to undergo apoptosis, as seen in Figure 8 C & D, there was an increased susceptibility to undergo apoptosis from baseline values that peaked during acute infection (week 3 p.i.) in the control animals with a similar increase in the JAK3 inhibitor group except that the peak was noted at week 6 post infection in these animals. Thereafter, the susceptibility to undergo apoptosis decreased to baseline values by 16 weeks post infection. These data suggest that the increased susceptibility to undergo apoptosis is a generalized function of initial mobilization but not linked to plasma or GIT viral loads since sustained high viral loads during chronic infection do not appear to influence CD95 associated in vitro apoptosis susceptibility.

### Effect of the JAK3 Inhibitor on SIV Specific Antibody and ADCC Responses

**Figure 9:**
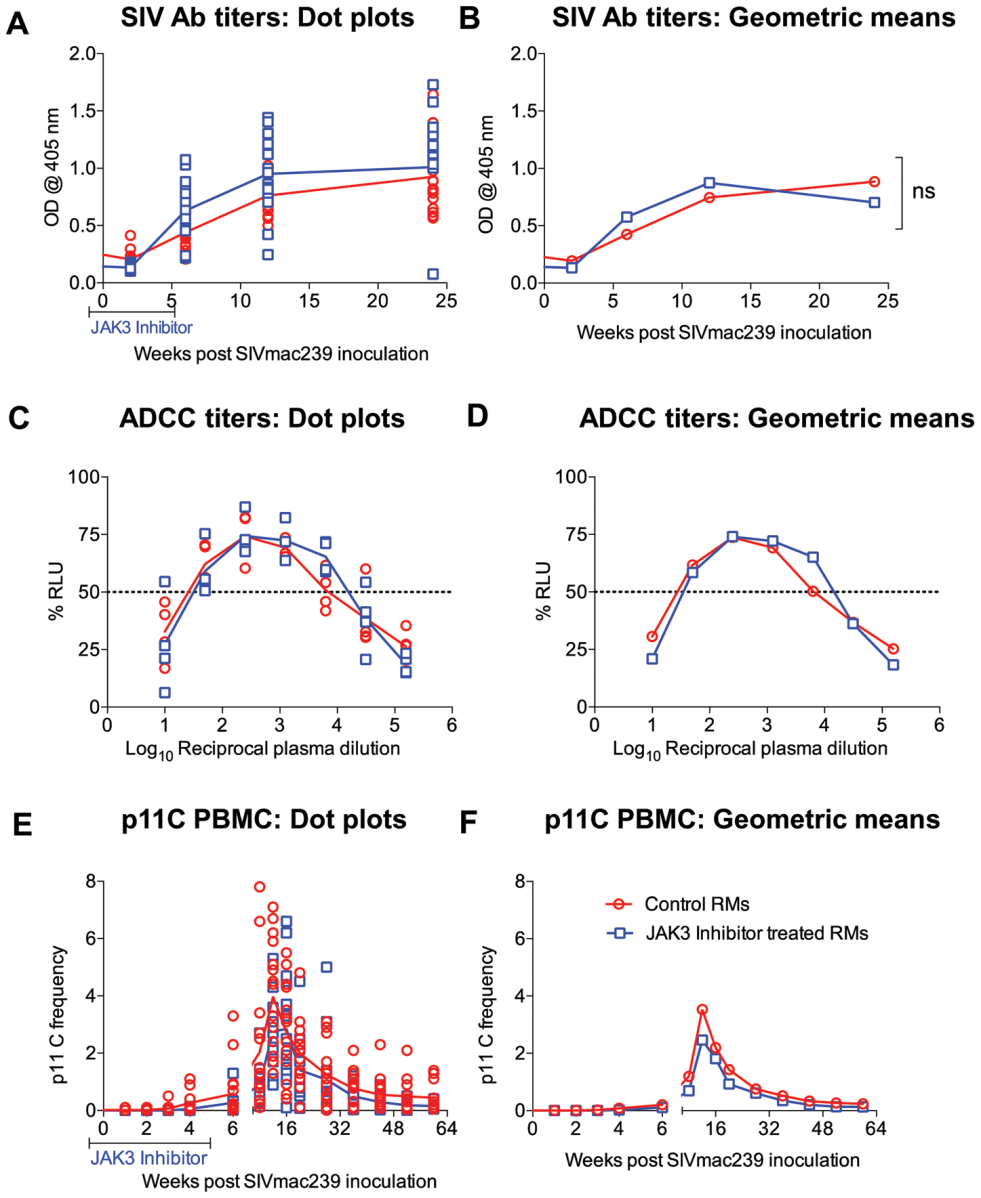
SIV specific antibody titers (A & B) calculated as O.D_450_ and ADCC titers calculated as IC_50_ values (C &D) in the plasma of the 2 groups of monkeys is illustrated as dot plots (A, C) or geometric means (B & D). In addition, the frequency of the p11C (C–M) tetramer^+^ cells as a function of the gated population of CD3^+^, CD8^+^ T cells are shown as a dot plot (E) or geometric means (F). The differences were not significant (n.s.).

Plasma from each of the 31 monkeys prior to (baseline) and at regular intervals following SIV infection were assayed for ELISA based anti-SIV IgG titers. Readily detectable anti-SIV specific antibodies were detectable as early as 3 weeks p.i. with peak levels at about 6 weeks post infection. While there was a trend for higher antibody levels in the JAK3 inhibitor group of animals, these differences were not statistically different (see Figure 9 A, B). Aliquots of the same plasma samples were assayed for ADCC activity. Results of this assay showed no statistically significant difference in the levels of ADCC antibodies in the animals that received the JAK3 inhibitor and the control animals (Figure 9 C, D) at each time point post infection. These data appear to suggest that depletion of NK cells during acute infection does not induce a significant effect on the SIV specific humoral response and the generation of functional ADCC antibodies.

### Effect of the JAK3 Inhibitor on p11C Peptide Specific Frequencies

Clearly one of the issues with the administration of the JAK3 inhibitor is whether such inhibitor administration has effects on other immune function besides NK cells since the JAK3/STAT-5a pathway affect all cell lineages that respond to the common gamma c chain utilizing cytokine receptors which include IL-2, 4, 7, 9, 15 and 21 [68]. In addition, while there is an element of specificity for JAK3 (1 nM, IC_50_), this is dependent on the dose since the IC_50_ for JAK1 and JAK2 is 112 nM and 20 nM, respectively [69]. However, it is important to note that a detailed pharmacokinetic study was performed by our laboratory in rhesus macaques and a dose was chosen that to a large extent predominantly depleted NK cells [44]. To address this issue of the effect of JAK3 inhibitor specificity, we deliberately chose all 35 monkeys that were Mamu-A01^+^ monkeys in the present study (see Table S1) so that we could monitor the effects of JAK3 inhibitor administration on the kinetics, frequency and function of p11C-tetramer^+^ cells. Results of such monitoring showed no statistical difference in the kinetics and frequencies of p11C-tetramer^+^ cells in the 2 groups of monkeys (Figure 9 E & F) suggesting that JAK3 inhibitor administration at the doses utilized has no detectable inhibitory effect on the generation of p11C peptide specific CD8^+^ T cell responses. Functional studies that included analysis of SIV peptide specific immune responses using the ICC and ELISPOT assays using pools of overlapping SIV env and gag peptides was also carried out on samples collected at the same time points post infection from the 2 group of animals. However, the data obtained did not appear to show statistical differences between the 2 groups of animals (data not shown for purposes of brevity). These data, as measured by these assays, seem to suggest that not only does the administration of the JAK3 inhibitor at least at the doses utilized fail to show any marked influence on the generation of antigen specific immune responses in SIV infected animals but that the depletion of NK cells also does not appear to influence these parameters. Studies of animals immunized with nominal antigens and administered the JAK3 inhibitor need to be performed to determine if our failure to detect effects of the JAK3 inhibitor was because we studied this in SIV infected animals.

## Discussion

The studies reported herein are the first to explore the effect of the administration of a JAK3 inhibitor on the pathogenesis of SIV infection during acute infection in nonhuman primates. One of the central issues with regards to the interpretation of data presented herein concerns the cell lineage targeting specificity of this JAK3 inhibitor and its general mechanisms of action. Briefly, while this JAK3 inhibitor (Tofacitinib) has been shown to inhibit not only JAK3 but to some extent also JAK2 and to a much lesser extent JAK1, it has a high degree of specificity for this family of kinases [70]. The expression of JAK3 is limited to lymphoid cells and JAK3 has been shown to associate intra-cellularly with the common γ chain (CD132) bearing cytokine receptors that include IL-2, 4, 7, 9, 15 and 21 and thus has the potential to inhibit the function of these cytokines and modulate both innate and adaptive immune responses [71]. The fact that JAK3 deficient mice exhibit profound T cell impairment and JAK3 deficient patients exhibit severe combined immunodeficiency [72], [73] has led to an intense effort to identify inhibitors with specificity for JAK3 as unique immunosuppressive therapeutic agents. These efforts led to the identification of Tofacitinib, which is one of the first JAK3 inhibitor to be used clinically. While initially Tofacitinib was used to prevent allograft rejection, it has since been utilized as an immunosuppressive agent and tested in patients with rheumatoid arthritis, Inflammatory bowel disease, psoriasis etc [74]. It should be noted that the in vivo effects of this JAK3 inhibitor appear to be highly dose dependent. Thus, at low doses, this JAK3 inhibitor was shown to enhance experimental autoimmune encephalitis but suppress collagen induced arthritis [75]. Thus, a considerable amount of effort was made by our lab to identify a dose that would achieve the goals of the studies described herein. However, just because we note that this dose predominantly depletes NK cells in vivo does not imply that this dose of the JAK3 inhibitor mediates its noted effect on viral loads by such NK cell depletion alone since the inhibition of other signaling pathways may also contribute to the observed results. Thus, a note of caution in the interpretation of the data presented herein.

The rhesus macaques included in the present study were subjected to MHC/KIR polymorphism analysis to ensure that the monkeys in the control and JAK3 inhibitor arms of the study were as evenly distributed as possible (see Table S1) to limit any role of host genetics on the study objectives. Detailed studies of the potential role of MHC/KIR polymorphisms in these and an additional large cohort of SIV infected rhesus macaques is currently in progress. Our lab has previously published results obtained on treating rhesus macaques with this same JAK3 inhibitor with the aim to study the potential role of JAK3 inhibitor sensitive cells during chronic SIV infection. This previous study included extensive in vitro and in vivo pharmacokinetic studies of the JAK3 inhibitor in rhesus macaques in attempts to define the optimum dosage to be utilized for the predominant depletion of the NK cell lineage [44]. Results from this previous study showed that at the doses utilized, the JAK3 inhibitor was highly effective in the depletion of the NK cell lineage and led to a transient but modest increase in plasma and pro-viral DNA load of the monkeys. Due to its ability to predominantly deplete NK cells in vivo (with the caveats described above) as shown by our study and a previous study in macaques [44], [45], and the documented potent ability of this JAK3 inhibitor to effectively inhibit IL-15 triggering particularly in the GIT [76]–[78], a cytokine critical for NK cell development and differentiation [79], it was reasoned to be an effective tool to study the in vivo role of JAK3 inhibitor including its effect on NK cell depletion during acute SIV infection. We were expecting that the major effects of the administration of the JAK3 inhibitor during acute infection would be targeting cells involved in mediating innate immune responses such as the NK cell lineage. However, as noted, we did not in fact note significant effects on plasma viral loads during acute infection although there did appear to be a significant increase in the GIT pro-viral DNA load (see Figure 1 A, B). It is possible that the inability to influence detectable changes in plasma viral load during acute infection was secondary to additional effects of the JAK3 inhibitor such as its ability to also decrease cell activation that is required for viral replication. Fortunately, we decided to continue to monitor both groups of animals and to our surprise found that the JAK3 inhibitor group of animals as compared to the control animals gradually developed markedly higher plasma (p<0.0001) and GIT viral loads during chronic infection (p<0.0001) (Figure 1 and 2). These findings clearly raise a series of questions. Thus, how does the administration of a JAK3 inhibitor during “acute” infection lead to such profound effects during “chronic” infection?. What is the tissue source for these high viral loads? What are the immunological mechanisms that promote such increased viral loads? How do we distinguish the effects of SIV infection alone from those mediated by the JAK3 inhibitor by itself? First of all, although no statistical difference in plasma viral loads were noted during acute infection, it is to be noted that there was a significant increase of pro-viral DNA load in the GIT of the JAK3 inhibitor group of animals at 1 week p.i. during acute infection (p<0.005) and during peak viremia (p<0.004). There was also a second wave of viremia within the GIT during chronic infection (Figure 2), a time period during which the CD4^+^ T cells are presumably massively depleted. Preliminary studies aimed at identifying the cell lineages that contribute to viremia during acute versus chronic infection within the GIT using FISH appear to implicate CD4^+^ T cells in both cases (data not shown). Whether these CD4^+^ T cells belong to the same subset remains to be defined. The discordance in plasma versus GIT viral loads suggest that the similar level of plasma viremia in the 2 groups of animals during acute infection must mean that increased viral replication in the control animals must occur at some other tissue site than the GIT. Unfortunately we did not monitor viral loads in any other lymphoid tissue during acute infection. Secondly, the fact that the administration of the JAK3 inhibitor did not completely eliminate the NK cells (75 to 80% depletion, Figure 3), prompted us to utilize the in vivo administration of a novel “primatized” anti-IL-15 monoclonal antibody (known to deplete NK cells in vivo) as a supplement to the JAK3 inhibitor to more efficiently deplete the NK cell lineage during “acute” SIV infection in a small group of 4 rhesus macaques (Group 3). It was reasoned that data from such more effective depletion of the NK cells might in concert with data derived using the JAK3 inhibitor alone (Group 2) may provide more refined clues as to the mechanisms involved. The previously optimized protocol utilized for the in vivo administration of anti-IL15 mAb was obtained courtesy of Dr. Afam Okoye (Oregon Health Sciences University, Beaverton, OR). As seen in Figure S5 C, such supplementation more effectively depleted the NK cell lineage (>99.9% depletion) that stayed maximally depleted until week 15. Thereafter, there was a gradual sequential replenishment of the subsets in order first by the CD16^−^/CD56^+^ subset (predominantly cytokine synthesizing NK cell subset) followed by the CD16^−^/CD56^−^, then the cytolytic CD16^+^/CD56^−^ subset and finally the CD16^+^/CD56^+^ subset (data not shown). This ordered sequence provides clues to the potential differentiation sequence of each of these NK cell subsets [18], [80], [81]. However, of concern was the finding that such supplemental anti-IL15 administration also led to the transient (nadir at week 4) depletion of an average of approximately 81.4, 79.3, and 86.5% of the total CD3^+^ T cells, CD4^+^ T cells and CD8^+^ T cells, respectively, with a gradual return to sub-baseline levels for each lineage by week 12–15 (see Figure S5 C). Thus, there was a more generalized decrease of multiple lymphoid cell subsets with such adjunct therapy consistent with a role of this cytokine in influencing both innate and adaptive immune responses [82]. The data obtained on these 4 animals thus needs to be interpreted within this context. Germane to the major thrust of these studies, as noted in Supplementary Figure S5 A & B, once again, while we failed to note any major difference in plasma viral loads between the controls (Group 1) and the animals that received the JAK3 inhibitor+anti-IL15 (Group 3), there was a significant difference between these Group 3 monkeys and those that received the JAK3 inhibitor alone (Group 2) monkeys during the early chronic infection at week 12–18 (p<0.001) time period. These data at face value indicate that the mechanisms that led to increased viral loads due to the JAK3 inhibitor alone were nullified by the supplementation with anti-IL15. We hypothesize that the cell lineage that promotes higher viral load in the Group 2 JAK3 inhibitor alone group of animals is depleted and/or dysregulated and is not replenished in the Group 3 animals. Data on the GIT viral loads on the other hand was similar in the Group 2 versus the Group 3 animals. Unfortunately such severe depletion of the NK cells and the other cell lineages during acute infection resulted in increased susceptibility of 2/4 monkeys to opportunistic infections and the animals had to be euthanized preventing us from studying the effects of such supplementation therapy for a prolonged time period during chronic infection. We did not include a control group of animals that received anti-IL15 administration alone during acute infection since such a study has already been conducted by the Picker lab [83]. Data from that study suggest that such acute depletion of NK cells with the in vivo administration of anti-IL15 mAb alone had no detectable effect on plasma viral loads. Of note, the findings reported herein markedly differ from studies that utilized mAb against CD8α for depletion of both CD8^+^ T cells and NK cells during acute infection [84], [85]. Results of that study led to fast progression of disease accompanied by severely diminished SIV specific immune response. Such differences in pathogenesis further supports the view that the JAK3 inhibitor does indeed target NK cells among the lymphoid cells. The fact that the in vivo administration of the anti-IL15 mAb coupled with the JAK3 inhibitor markedly depletes multiple cell lineages during acute infection, unfortunately, made it difficult for us to better define the potential mechanisms for increased plasma viralloads due to JAK3 inhibitor administration.

As stated above, an extensive series of phenotypic and functional study of blood and GIT samples were conducted on each of the 35 animals that were included in this study in efforts to identify clues for the increased plasma viral loads in the Group 2 animals during chronic infection. Some of the striking differences included the finding that both the NKG2a^+^ and NKG2a^−^ subset of cells in the GIT were not only depleted by SIV infection alone but were also more severely depleted in the SIV infected animals that received the JAK3 inhibitor (Figure 7 E–H). This cell lineage was never replenished even in animals with low to modest plasma and GIT pro-viral DNA loads in both groups of animals. This was also true for the NKp44^+^ subset of NK cells also termed innate lymphoid cells (ILC's) and confirms the findings of Reeves et al [17]. This irreversible depletion of the NKG2a^+^ cells suggest that while SIV infection alone leads to such depletion, the addition of the JAK3 inhibitor may additionally deplete a subset whose absence leads to the observed increases in the plasma viral load during chronic infection. This finding has important implications for current therapeutic protocols that involve the clinical use of this JAK3 inhibitor for the therapy of rheumatoid arthritis, a variety of gut associated diseases including Crohn's, ulcerative colitis and IBD [51], [86]–[89]. It is to be noted that NK cells express the gut homing hetero-dimeric integrin α4β7, which cells utilize to home to the GIT [90]–[93]. It is thus possible that SIV infection inhibits the upregulation of this homing receptor that, in turn, accounts for the failure of cells to home to the GIT. It is to be noted that following homing to the GIT, there is marked down regulation of α4β7 expression and the cells appear to switch to expressing αEβ7 and that this property could have been influenced by the JAK3 inhibitor. On the other hand, it could be that SIV infection dysregulates the physiologically normal expression of MAdCAM-1, the cognate ligand for α4β7 by cells of the GIT contributing to the failure of these NK cells to anchor within the GIT. A preliminary study involving infusion of CFSE labeled autologous in vitro expanded α4β7 expressing NKG2a^+^ cells in normal rhesus macaques suggest that a significant number of these cells indeed home to the GIT. A similar study in SIV infected animals needs to be performed that may help distinguish whether the failure to home to the GIT is due to aberrant α4β7 expression, MAdCAM-1 expression, or both. The point remains, as stated above, that this cell lineage remains depleted in the GIT of SIV infected animals that is further exacerbated with the JAK3 inhibitor. It is reasoned that a JAK3 inhibitor sensitive cell lineage plays a contributory role within the GIT that could be via the TIM-3/Galectin-9 pathway [94] and its absence leads to chronic GIT pathology contributing to higher viral loads. It is important in this regard to determine whether this effect of irreversible NK cell depletion is secondary to SIV infection or due to the administration of the JAK3 inhibitor. In a previous study, a group of 6 rhesus macaques were administered the same dosing regimen of the JAK3 inhibitor (although a different batch of this inhibitor was utilized). Results of this study showed that at the doses of JAK3 utilized, while there were decreases in the levels of both CD4^+^ and CD8^+^ T cells in the blood, which were maximal at 3–4 weeks, the major depleting effect was in the frequency of CD3^−^/CD8^+^/NKG2a^+^ (NK cells) with minimal effects on pDC's (see Figure S6). These decreases in each of the cell lineages were transient and returned back to baseline levels by week 6–8. Of significance was the finding that whereas the administration of the JAK3 inhibitor depleted the NKG2a^+^ cells within the GIT (see Figure S7), this cell lineage also returned back to baseline values by week 6–8. These data suggest that the irreversible depletion of the NKG2a^+^ cells in the SIV infected animals (Figure 7 E–H) was to a large extent likely due to SIV infection.

The next issue of interest was the observation of a dichotomy between GIT viral loads and plasma viral loads in the 2 groups of animals. Thus, similar plasma viral RNA loads in the 2 groups of monkeys but distinctly higher GIT pro-viral DNA loads in the JAK3 inhibitor monkeys during acute infection could be due to a higher frequency of pro-viral DNA bearing cells that are not replicating virus which could be due to the inhibition of cell activation function of the JAK3 inhibitor. The issue that is difficult to explain is the high pro-viral DNA load in the GIT of the 2 groups of animals during chronic infection even when there is a dearth of CD4^+^ T cells within these tissues. It is possible that the pro-viral DNA load during chronic infection is secondary to non-specific viral entrapment and/or due to other than CD4^+^ T cells that are contributing to this increased pro-viral DNA load. Although the major source of the pro-viral DNA load appeared to be CD4^+^ T cells, we did not examine subsets of this cell lineage that were the source of the pro-viral DNA load during chronic infection, a study we need to perform in the future.

We were also intrigued by the finding of the increased levels of total and, in particular the naive and to some extent the central memory CD4^+^ T cells during chronic infection in the JAK3 inhibitor group of animals as compared with the control animals (see Figure 5 E–F) despite very high plasma and GIT viral loads (see Figure 1 & 2). It is reasonable to assume that this is secondary to either a difference in the CD4^+^ T cell subset targeted by the virus, due to longer half life of the select subsets, due to differences in the circulation/mobilization of the subsets or lower levels of elimination of infected cells via ADCC in the JAK3 inhibitor animals. Differences in the CD4^+^ T cells that are the target of SIV infection have been recorded in the case of natural versus non-natural hosts of SIV infection [95], [96]. We did monitor for the frequencies of CD4^+^ T cell subsets that express Ki67^+^ and while there was a trend of increased levels of Ki67^+^ CD4^+^ T cells in the JAK3 inhibitor group of animals, there was a high degree of variability and therefore difficult to ascribe the differences being secondary to the turnover rate of the subsets. In vitro analysis for susceptibility to SIV infection of Con-A stimulated PBMCs during this chronic infection period from the 2 groups of animals with the same input virus stock also failed to show any differences but we recognize the limitation of this type of analysis. The cytokines that are potentially involved in contributing to the increases in these subsets of CD4^+^ T cells is currently under study. We also examined the data on the frequencies of CD4^+^ T cells that express CCR5 and α4β7 and levels of CD4^+^/CD8^+^ Tregs at baseline since these markers have been shown previously to contribute to and/or be preferential targets of SIV infection [97]–[99]. While there did not appear to be any major difference in the frequencies of CCR5 expressing CD4^+^ T cells in the blood and GIT of the 2 group of animals, there clearly appeared to be an association between high frequencies of α4β7^hi^ expressing CD4^+^ T cells in the GIT biopsies at baseline with high peak viral loads but this was evident in both groups of animals (data not shown). These findings on α4β7^hi^ expressing CD4^+^ T cells and higher viral loads confirm the previous reported data [91]. We also monitored the frequencies of CD4^+^ and CD8^+^ Tregs in the blood and GIT in a sub-group of animals from the 2 groups but failed to show any significant differences, at least in the frequencies of these regulatory T cell subsets. However, there maybe differences in the function of such subsets that we have not yet analyzed. We also analyzed the frequencies of infected cells in the PBMC of the 2 groups of animals using a conserved SIVgag probe and in situ hybridization techniques but failed to identify any significant differences (range 0.01 to 0.001 ISH^+^ cells). However, we recognize that the differences could be in the frequencies of infected cells within the local lymph nodes and/or other tissues of the 2 groups of animals.

It is important to note that we did not find any differences in the frequencies and/or kinetics of p11c tetramer^+^ cells generation in the 2 groups of animals (see Figure 9 E & F). These data suggest that the administration of the JAK3 inhibitor did not appreciably influence the induction of SIV antigen specific CD8^+^ T cell immune responses (at least using this measure) and thus differences in the viral load during chronic SIV infection in the JAK3 inhibitor group of animals could not be reasoned to be secondary to a delay in the kinetics and generation of virus specific immune responses. Thus, although NK cells are presumed to influence the generation of adaptive immune responses, most of these data are acquired using murine models and suggest a note of caution on extrapolating data from the murine model to other species. Polychromatic cytokine inducing antigen specific immune function of either total PBMC and/or p11C tetramer^+^ CD8^+^ T cells performed on a subset of PBMC samples from the 2 group of animals failed to show any marked differences in the profile (data not shown). These findings suggest that we have yet to identify a cellular immune based assay that truly distinguishes responses in the Group 1 and 2 animals in the present study that is also true in general for identifying protective immune responses in the SIV infected model of HIV-1 infection.

One of the interesting findings in the anti-IL15+JAK3 inhibitor group of animals was that while these 4 animals appear to show titers of anti-SIV antibody responses similar to those seen in the group 1 and 2 animals, they each had either a low or an undetectable titer of SIV specific ADCC antibody response (data not shown). These data suggest that the administration of the anti-IL15 mAb clearly selectively influences the generation of ADCC functional anti-SIV antibodies but not total anti-SIV antibody responses. The important role and limitations of such ADCC antibodies in HIV infection [100]–[102] and the problems associated with inducing such antibodies has recently been highlighted [103]. It is to be noted that there was a significant increase in the absolute number of B cells in the JAK3 inhibitor group of animals at weeks 16–20 p.i. (See Figure 6 C & D). The significance of this finding is not readily apparent.

The finding that JAK3 inhibitor administration inhibits the mobilization of pDCs during acute infection (see Figure 6 E & F), may also contribute to the increased plasma viral loads during chronic infection. Thus, it is recognized that pDCs are the major source of IFN-α [63], [64], [104], [105] and that type I interferon could lead to increased synthesis of other innate immune factors such as tetherin that inhibit viral release and also are known to sense viral infection and activate NFkB anti-inflammatory pathway contributing to control of virus replication [106]. The lack of their mobilization in the blood and localization to the GIT may provide the window period that leads to increased plasma viral loads during chronic infection in the JAK3 inhibitor group of animals. During chronic infection, however, there is a similar accumulation of high levels of pDCs in the GIT associated with a depletion of the NK cell lineages suggesting that the early kinetics of pDC mobilization to the GIT and depletion of NK cells maybe the key to such differences in viral loads during chronic infection in the JAK3 inhibitor group of animals. Cross talk between NK cells and pDCs has been shown to play an important role in the generation and regulation of anti-viral immune responses including HIV-1 infection [107]–[110]. Thus, the kinetics of such interactions may influence the quality of the anti-SIV response that is generated that contributes to the high viral loads in the JAK3 inhibitor group of animals.

These data, in concert suggest that the administration of the JAK3 inhibitor has both negative and positive effects that together contribute to the higher viral loads during chronic infection in SIV infected rhesus macaques. The negative effects include the inhibition of pDC mobilization from the periphery to the GIT during acute infection, the irreversible depletion of both the NKG2a^+^ and the NKG2a^−^ subset of NK cells within the GIT, and a depletion of NK cell function. The positive effects include the selective maintenance of B cells and the naïve and central memory CD4^+^ T cells during chronic SIV infection. These findings are not trivial given the fact that this JAK3 inhibitor is being utilized as one of the major forms of therapy for a number of GIT diseases and in rheumatoid arthritis. A more detailed study aimed at distinguishing the immunological events that are mediated by the JAK3 inhibitor that are unique to SIV infection and those that are a general effect of the JAK3 inhibitor appear justified.

## Supporting Information

Figure S1 The overall protocol utilized for this study is depicted. There were 3 groups of rhesus macaques included in this study. Group 1 was the control group (n = 16), group 2 were the animals that received the JAK3 inhibitor orally (n = 15) starting day −6 until day 28 post infection (35 days) and group 3 animals (n = 4) received the same course of the JAK3 inhibitor as group 2 and in addition received the “primatized” anti-IL15 mAb starting on day −3 (20 mg/kg) followed by bi-weekly doses of 10 mg/kg intravenously for a total of 15 doses. The days of blood sampling and GIT tissue biopsy procurement are illustrated.
(TIF)

Figure S2 Representative profile of the gating strategies utilized to define the frequencies and absolute numbers of CD4^+^ T cells and CD8^+^ T cell subsets is illustrated.
(TIF)

Figure S3 Representative profile of the gating strategies utilized to define the frequencies and absolute numbers of NK cell and its subsets is illustrated.
(TIF)

Figure S4 Representative profile of the gating strategies utilized to define the frequencies and absolute numbers myeloid and plasmacytoid dendritic cells is illustrated.
(TIF)

Figure S5 Aliquots of the plasma from the 4 monkeys in Group 3 were analyzed for levels of virus and the data (Log10 vRNA copies/ml) for each of the 4 animals is illustrated in (A). For comparison, the geometric mean levels of plasma viremia for all 3 groups (16 in group 1, 15 in group 2 and 4 in group 3) are illustrated in (B). While there was no difference between the levels noted in samples from group 1 versus group 3 animals, there was a clear difference (p<0.001) in plasma viral loads between group 2 and group 3 animals at weeks 12–18. The absolute numbers of total CD3^+^ T cells, CD4^+^ T cells, CD8^+^ T cells, and CD3^−^, CD8a^+^, NKG2a^+^ cells in the PBMC of the 4 animals in Group 3 are illustrated (C) to emphasize the depletion of the multiple cell lineages. The subsets were also analyzed but not shown for brevity.
(TIF)

Figure S6 Aliquots of PBMCs from 6 normal rhesus macaques that were administered the same dose regimen of the JAK3 inhibitor (20 mg/kg daily orally starting day −6 until day 28) were analyzed for the absolute numbers of various lymphoid cell subsets. The dot plots (A, C, E and G) and geometric means (B, D, F & H) for the absolute values obtained for CD4^+^ T cells (A &B), CD8^+^ T cells (C &D), NKG2a^+^ cells (E & F) and plasmacytoid dendritic cells (G & H) are illustrated. Please note that the major depletion noted was for the NKG2a^+^ cells, (p<0.0001).
(TIF)

Figure S7 Aliquots of gastro-intestinal tissue biopsy procured lymphoid cells from the same 6 animals as described under Figure S3 were analyzed for the frequencies of various lymphoid cells on the gated population of CD45^+^ cells. The dot plots (A, C, E, G & I) and the geometric means (B, D, F, H & J) for the frequencies of CD4^+^ T cells (A & B), CD8^+^ T cells (C & D), NKG2a^+^ cells (E & F), NKG2a^−^ cells (G & H) and pDCs (I & J) are illustrated. Once again, please note that the major depletion that was seen was for the NKG2a^+^ cells, (p<0.0001).
(TIF)

Table S1 Each of the 31 animals included in this study was subjected to MHC and KIR typing as described in the methods section and a summary of the frequencies of MHC and KIR types in the control and the JAK3 treated groups of animals is described.
(TIFF)

Table S2 Detailed results of the MHC and KIR typing of individual animals in the control and JAK3 treated group is described.
(TIFF)
