# Supplementary figures and images for: In Vivo Administration of a JAK3 Inhibitor during Acute SIV Infection Leads to Significant Increases in Viral Load during Chronic Infection
Source: PLoS Pathog. 2014 Mar 6;10(3):e1003929. doi: 10.1371/journal.ppat.1003929 (PMC3946395; doi:10.1371/journal.ppat.1003929)

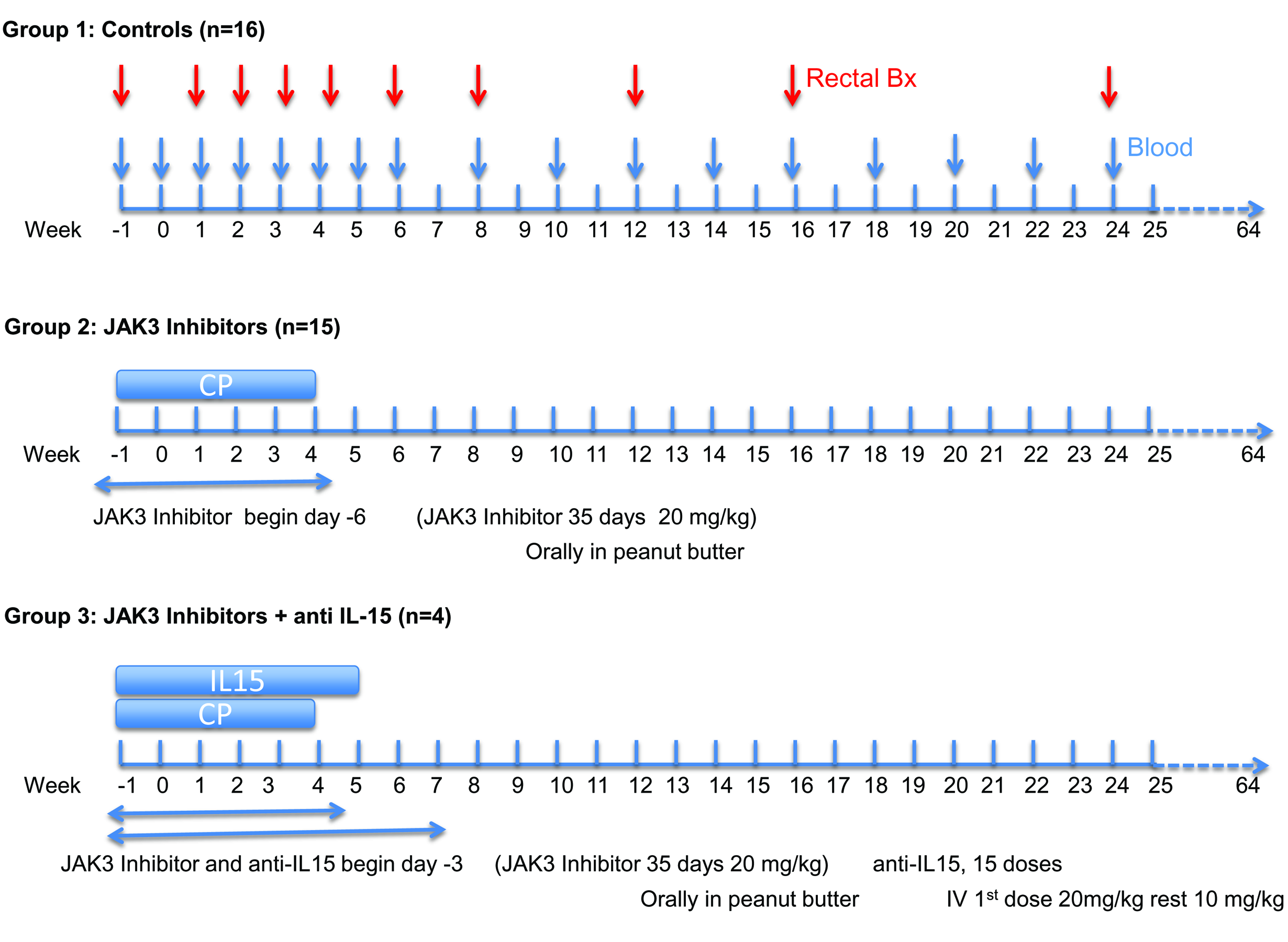

Supplement: Figure S1 — The overall protocol utilized for this study is depicted. There were 3 groups of rhesus macaques included in this study. Group 1 was the control group (n = 16), group 2 were the animals that received the JAK3 inhibitor orally (n = 15) starting day −6 until day 28 post infection (35 days) and group 3 animals (n = 4) received the same course of the JAK3 inhibitor as group 2 and in addition received the “primatized” anti-IL15 mAb starting on day −3 (20 mg/kg) followed by bi-weekly doses of 10 mg/kg intravenously for a total of 15 doses. The days of blood sampling and GIT tissue biopsy procurement are illustrated. (TIF) [file ppat.1003929.s001.tif]

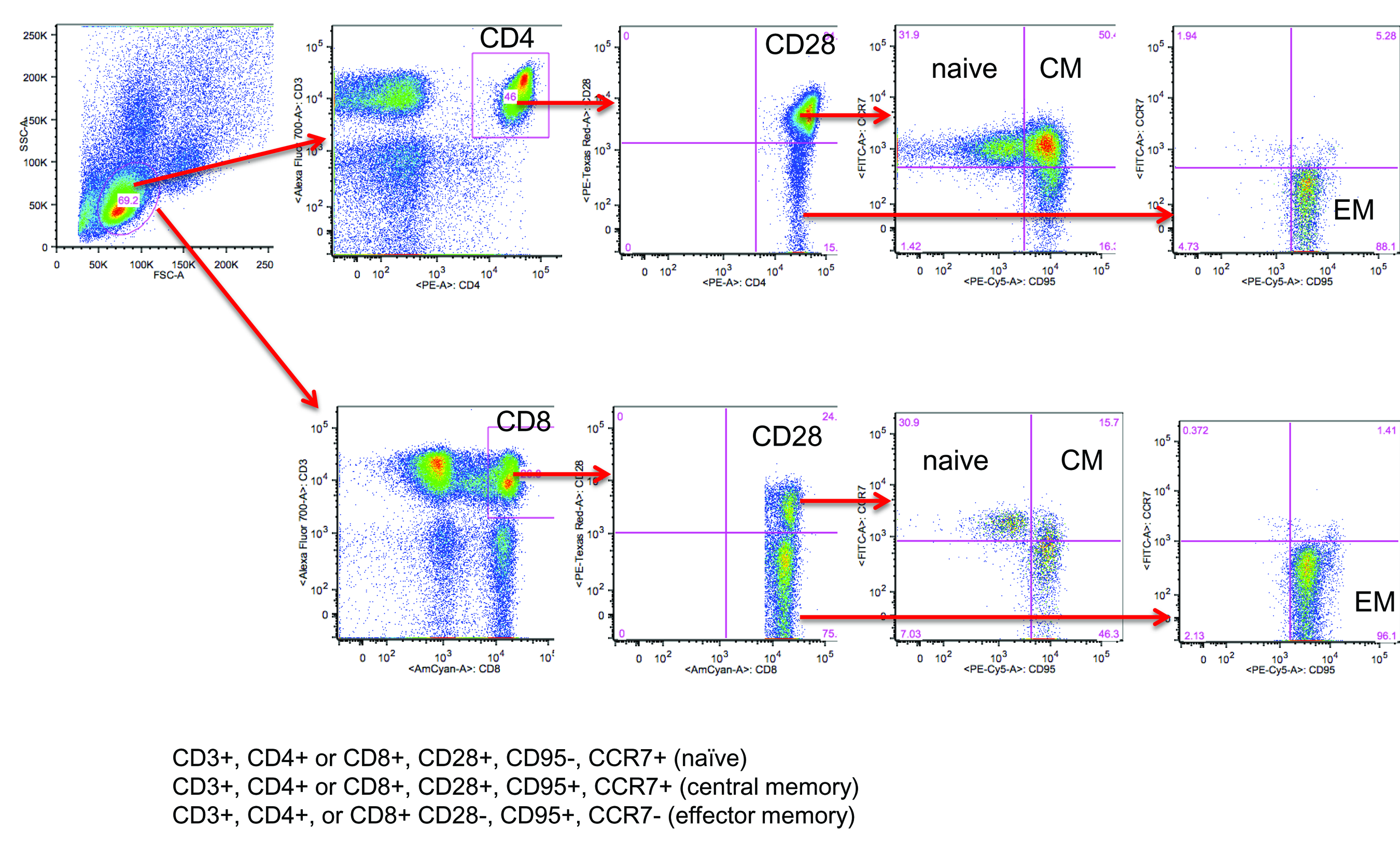

Supplement: Figure S2 — Representative profile of the gating strategies utilized to define the frequencies and absolute numbers of CD4+ T cells and CD8+ T cell subsets is illustrated. (TIF) [file ppat.1003929.s002.tif]

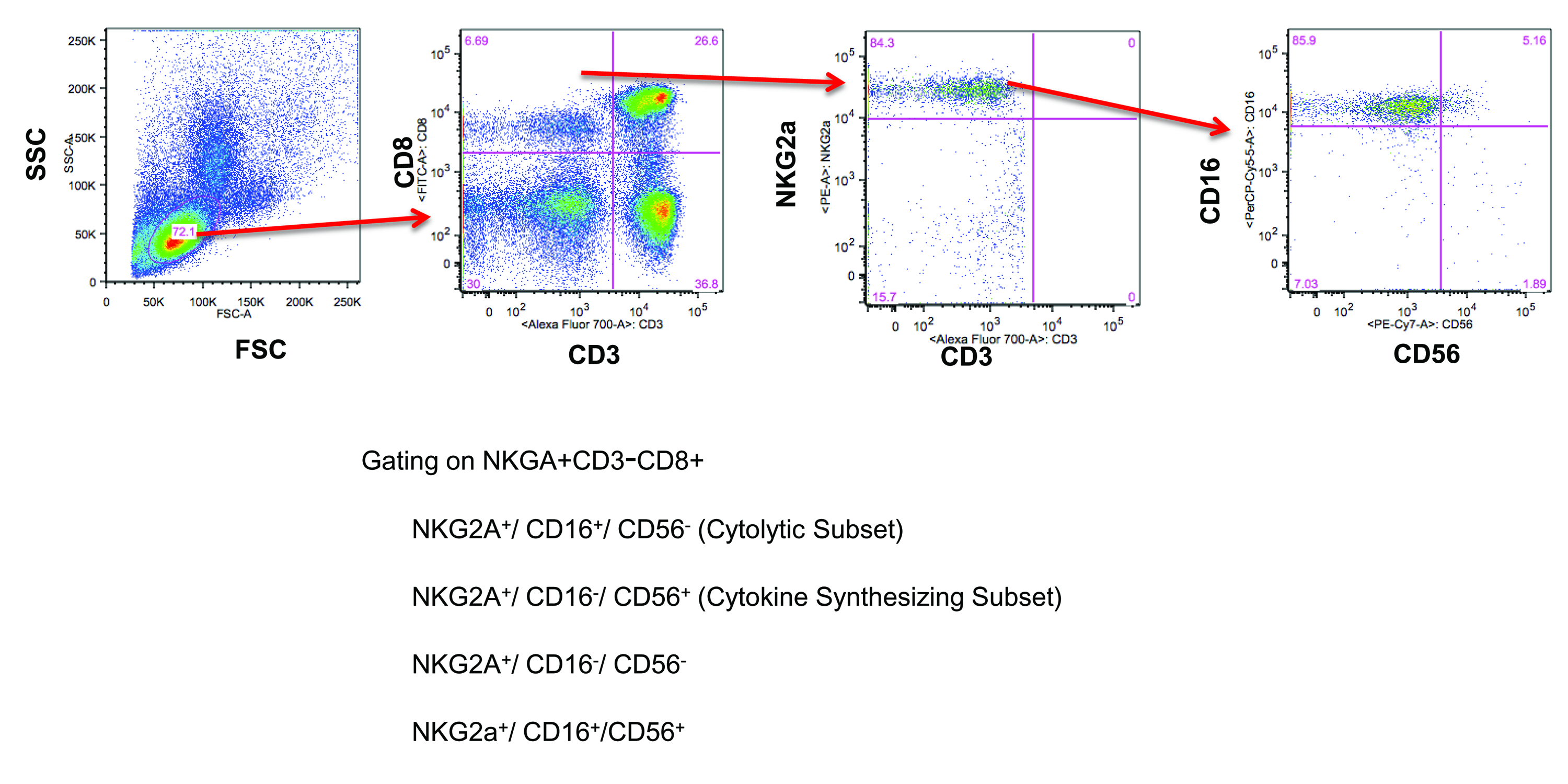

Supplement: Figure S3 — Representative profile of the gating strategies utilized to define the frequencies and absolute numbers of NK cell and its subsets is illustrated. (TIF) [file ppat.1003929.s003.tif]

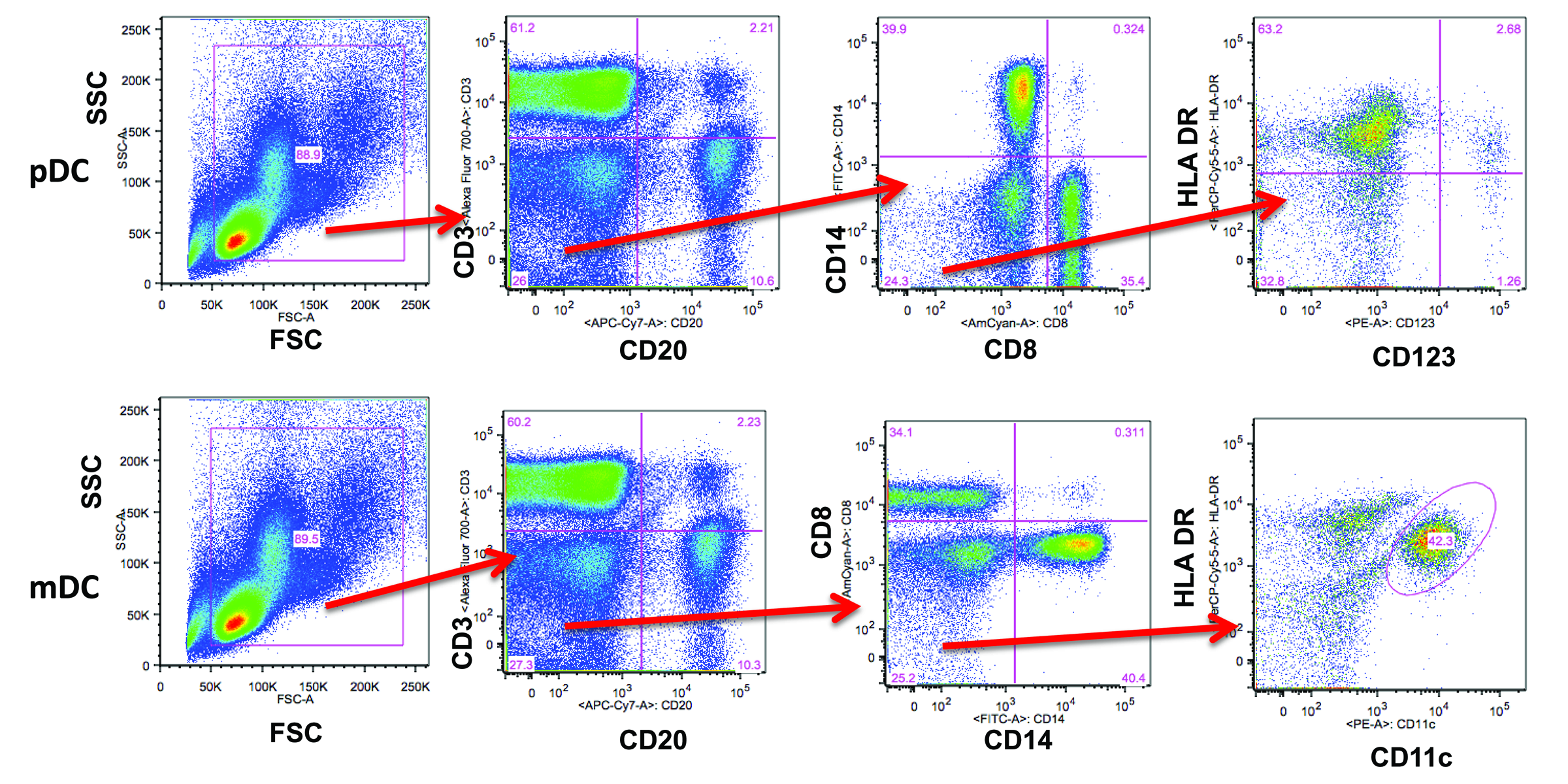

Supplement: Figure S4 — Representative profile of the gating strategies utilized to define the frequencies and absolute numbers myeloid and plasmacytoid dendritic cells is illustrated. (TIF) [file ppat.1003929.s004.tif]

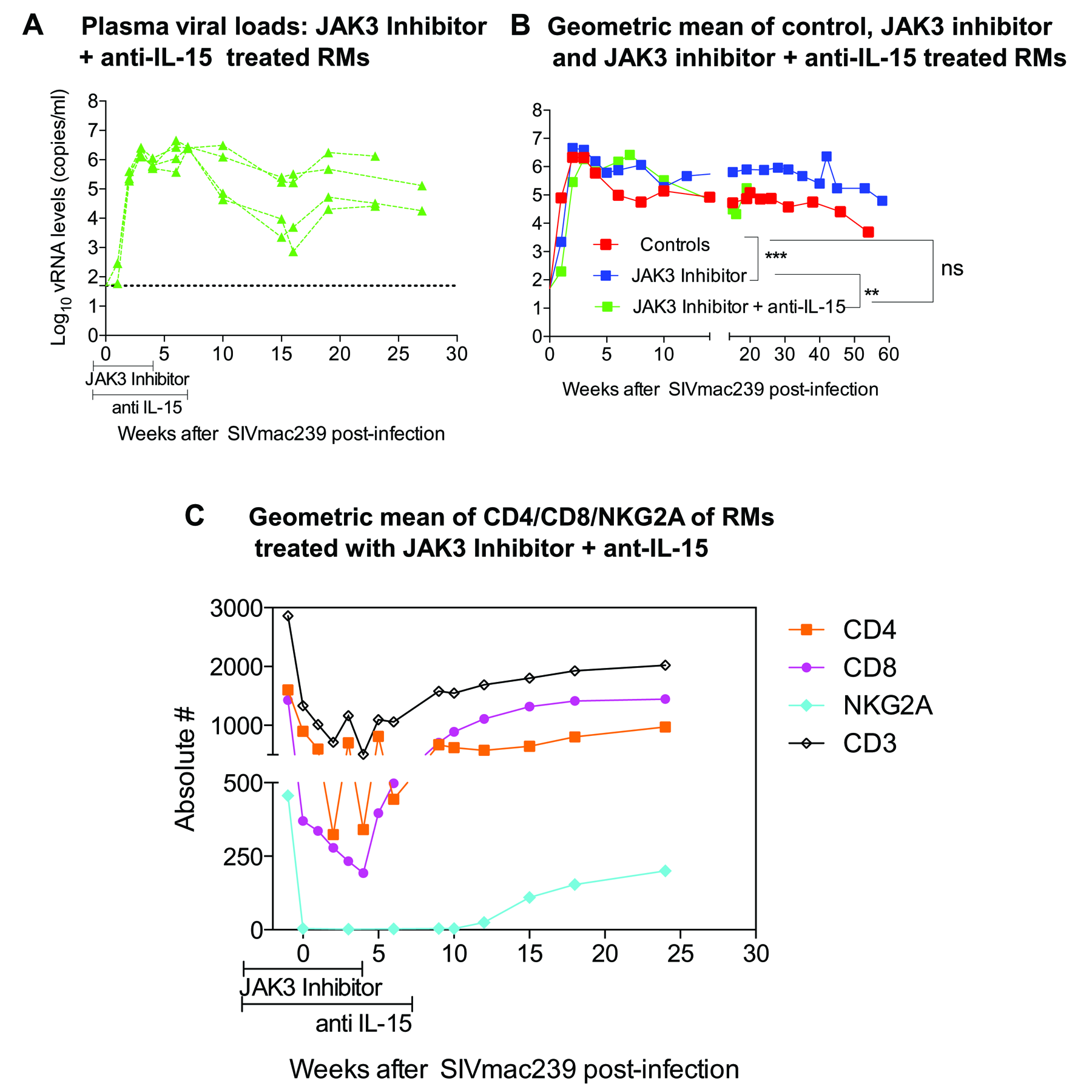

Supplement: Figure S5 — Aliquots of the plasma from the 4 monkeys in Group 3 were analyzed for levels of virus and the data (Log10 vRNA copies/ml) for each of the 4 animals is illustrated in (A). For comparison, the geometric mean levels of plasma viremia for all 3 groups (16 in group 1, 15 in group 2 and 4 in group 3) are illustrated in (B). While there was no difference between the levels noted in samples from group 1 versus group 3 animals, there was a clear difference (p<0.001) in plasma viral loads between group 2 and group 3 animals at weeks 12–18. The absolute numbers of total CD3+ T cells, CD4+ T cells, CD8+ T cells, and CD3−, CD8a+, NKG2a+ cells in the PBMC of the 4 animals in Group 3 are illustrated (C) to emphasize the depletion of the multiple cell lineages. The subsets were also analyzed but not shown for brevity. (TIF) [file ppat.1003929.s005.tif]

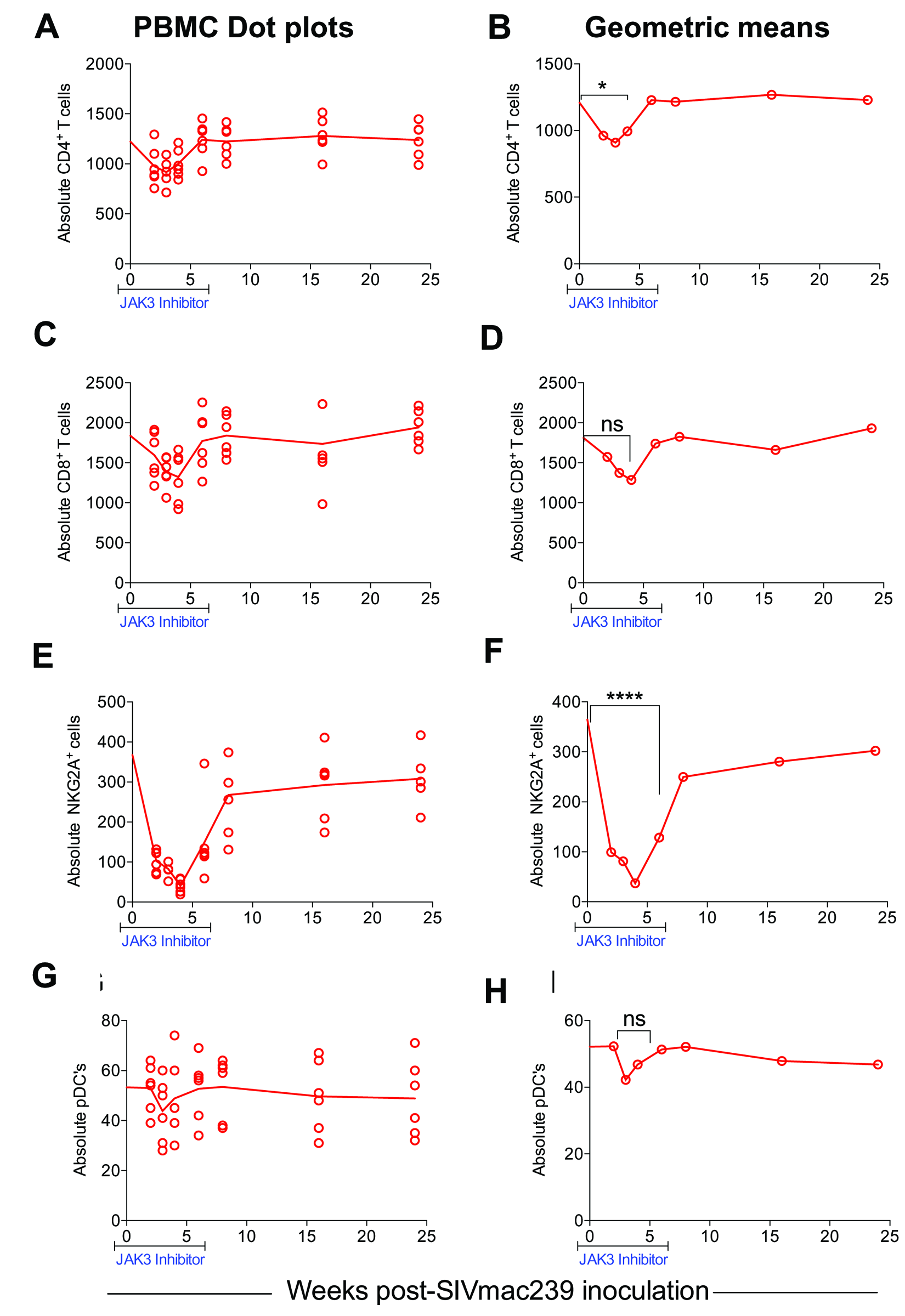

Supplement: Figure S6 — Aliquots of PBMCs from 6 normal rhesus macaques that were administered the same dose regimen of the JAK3 inhibitor (20 mg/kg daily orally starting day −6 until day 28) were analyzed for the absolute numbers of various lymphoid cell subsets. The dot plots (A, C, E and G) and geometric means (B, D, F & H) for the absolute values obtained for CD4+ T cells (A &B), CD8+ T cells (C &D), NKG2a+ cells (E & F) and plasmacytoid dendritic cells (G & H) are illustrated. Please note that the major depletion noted was for the NKG2a+ cells, (p<0.0001). (TIF) [file ppat.1003929.s006.tif]

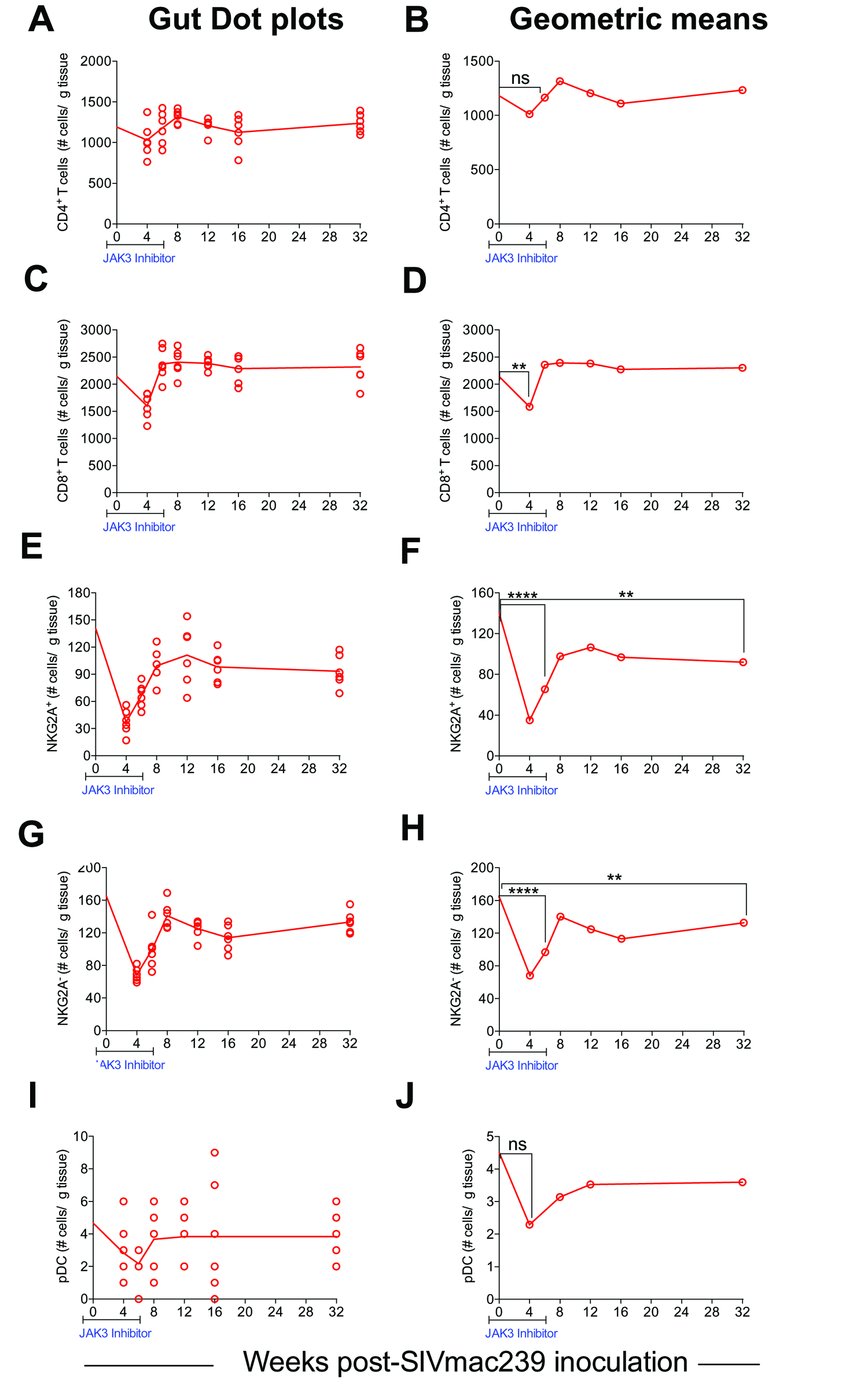

Supplement: Figure S7 — Aliquots of gastro-intestinal tissue biopsy procured lymphoid cells from the same 6 animals as described under Figure S3 were analyzed for the frequencies of various lymphoid cells on the gated population of CD45+ cells. The dot plots (A, C, E, G & I) and the geometric means (B, D, F, H & J) for the frequencies of CD4+ T cells (A & B), CD8+ T cells (C & D), NKG2a+ cells (E & F), NKG2a− cells (G & H) and pDCs (I & J) are illustrated. Once again, please note that the major depletion that was seen was for the NKG2a+ cells, (p<0.0001). (TIF) [file ppat.1003929.s007.tif]

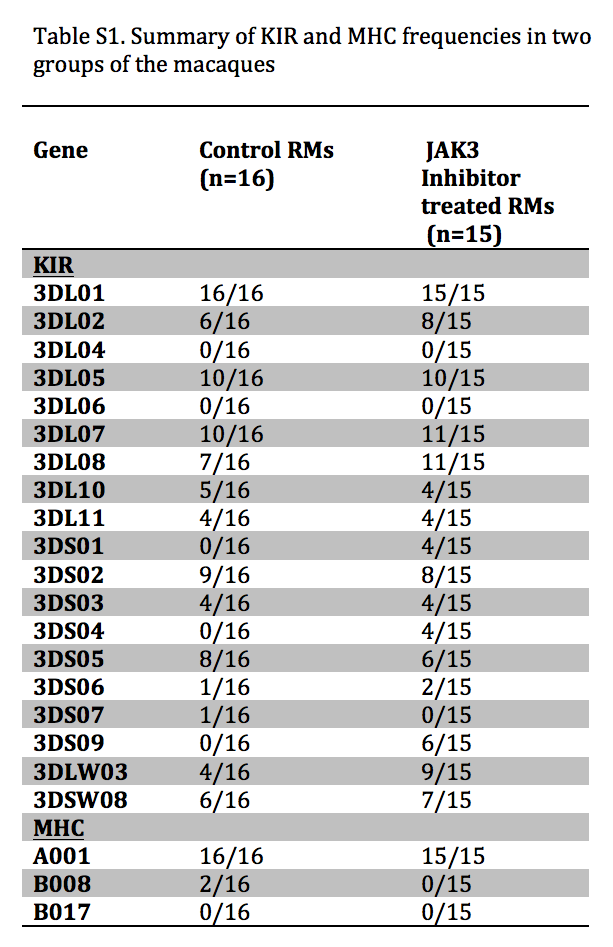

Supplement: Table S1 — Each of the 31 animals included in this study was subjected to MHC and KIR typing as described in the methods section and a summary of the frequencies of MHC and KIR types in the control and the JAK3 treated groups of animals is described. (TIFF) [file ppat.1003929.s008.tiff]

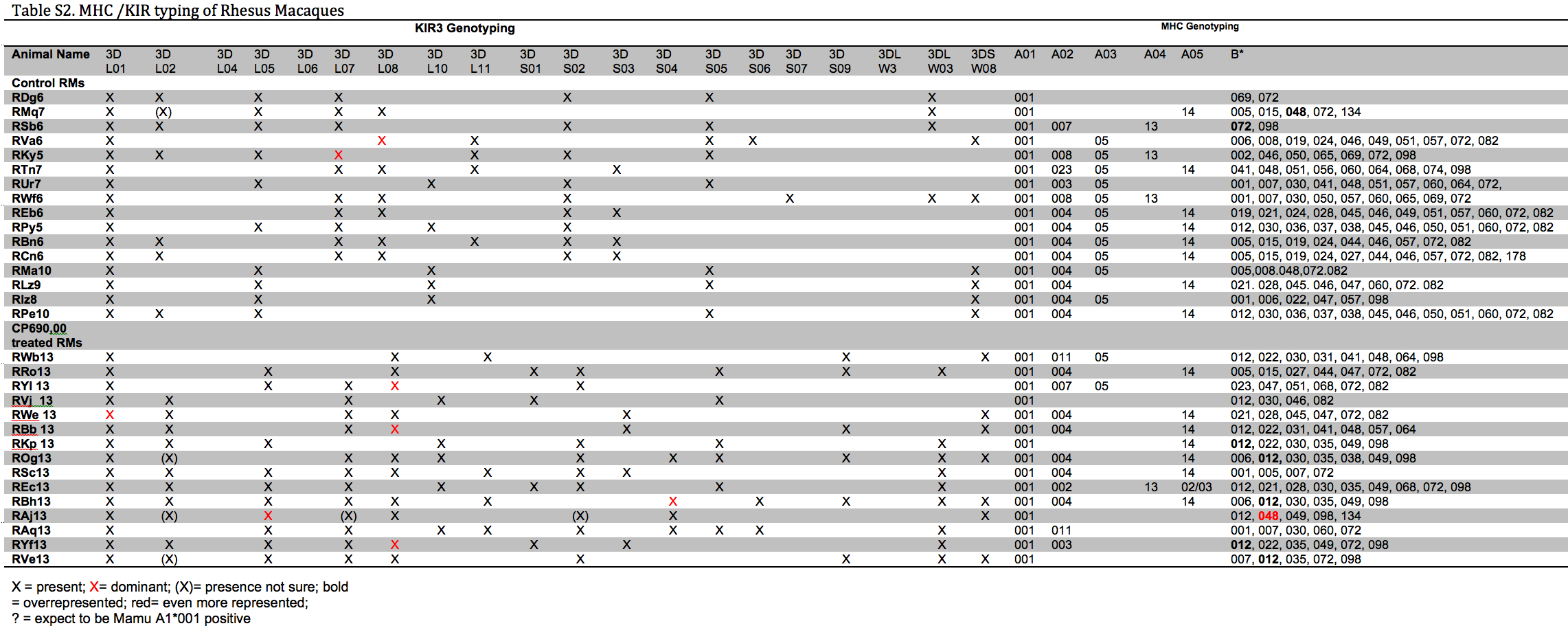

Supplement: Table S2 — Detailed results of the MHC and KIR typing of individual animals in the control and JAK3 treated group is described. (TIFF) [file ppat.1003929.s009.tiff]
